# Total synthesis of (+)-gelsemine via an organocatalytic Diels–Alder approach

**DOI:** 10.1038/ncomms8204

**Published:** 2015-05-21

**Authors:** Xiaoming Chen, Shengguo Duan, Cheng Tao, Hongbin Zhai, Fayang G. Qiu

**Affiliations:** 1 State Key Laboratory of Applied Organic Chemistry, Lanzhou University, Lanzhou 730000, China; 2 Laboratory of Molecular Engineering, and Laboratory of Natural Product Synthesis, Guangzhou Institute of Biomedicine and Health, Chinese Academy of Sciences, 190 Kaiyuan Boulevard, The Science Park of Guangzhou, Guangzhou 510530, China

## Abstract

The structurally complex alkaloid gelsemine was previously thought to have no significant biological activities, but a recent study has shown that it has potent and specific antinociception in chronic pain. While this molecule has attracted significant interests from the synthetic community, an efficient synthetic strategy is still the goal of many synthetic chemists. Here we report the asymmetric total synthesis of (+)-gelsemine, including a highly diastereoselective and enantioselective organocatalytic Diels–Alder reaction, an efficient intramolecular trans-annular aldol condensation furnishing the prolidine ring and establishing the configuration of the C20 quaternary carbon stereochemical centre. The entire gelsemine skeleton was constructed through a late-stage intramolecular S_N_2 substitution. The enantiomeric excess of this total synthesis is over 99%, and the overall yield is around 5%.

## Abstract (web summary)

Gelsamine is a naturally occurring alkaloid, whose complex structure makes it an interesting target for total synthesis. Here, the authors report an enantioselective synthesis of (+)-Gelsemine in 5% overall yield, with an enantioselective organocatalytic Diels–Alder reaction as one of the key steps.

## Graphical abstract

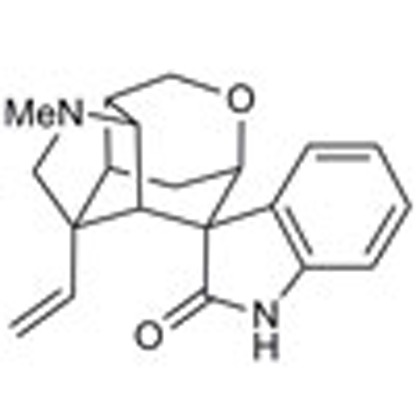

Although gelsemine was isolated^1^ in as early as 1876 from *Gelsemium Sempervirens* Ait., its structure was not determined until 1959 by means of nuclear magnetic resonance (NMR) spectroscopic techniques^2^,^3^ and X-ray crystallographic analysis^4^. This indole alkaloid contains a hexacyclic cage structure and seven contiguous chiral carbon centres (Fig. 1). The complex chemical structures of gelsemine and other members of the alkaloid family^5^,^6^,^7^,^8^ have attracted considerable attention from synthetic chemists. So far, in addition to the many synthetic efforts^9^,^10^,^11^,^12^,^13^,^14^,^15^,^16^,^17^,^18^,^19^,^20^,^21^,^22^,^23^,^24^,^25^,^26^,^27^,^28^,^29^,^30^,^31^,^32^,^33^,^34^,^35^,^36^,^37^, there are eight total syntheses reported in the literature^38^,^39^,^40^,^41^,^42^,^43^,^44^,^45^,^46^,^47^,^48^,^49^ (Fig. 2), two of which are asymmetric^44^,^48^. Although gelsemine was thought to have no particular biological activities, a recent report indicated that gelsemine exhibited potent and specific antinociception in chronic pain by acting at the three spinal glycine receptors^50^. Besides, gelsemine was nonaddictive, indicating that the mechanism of its action is different from that of morphine. The complex structure and the potential medicinal applications of gelsemine prompted us to initiate a more efficient enantioselective total synthesis.

Herein we wish to report a 12-step, highly enantioselective organocatalytic total synthesis of (+)-gelsemine.

## Results

### Retrosynthetic analysis

**Figure 3:**
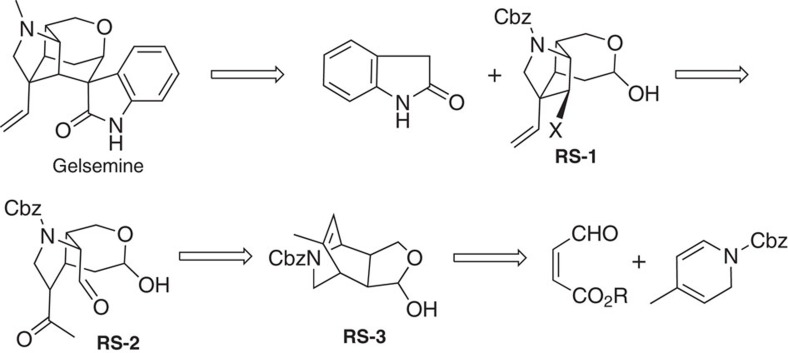
Retrosynthetic analysis of gelsemine. In principle, gelsemine may be constructed from oxindole and intermediate RS-1, where X is a leaving group. After a few transformations, RS-1 may be synthesized from intermediate RS-2, which inturn may be obtained from RS-3 following several reaction steps including ozonolysis. Finally, RS-3 may be synthesized from readily accessible starting materials.

**Figure 4:**
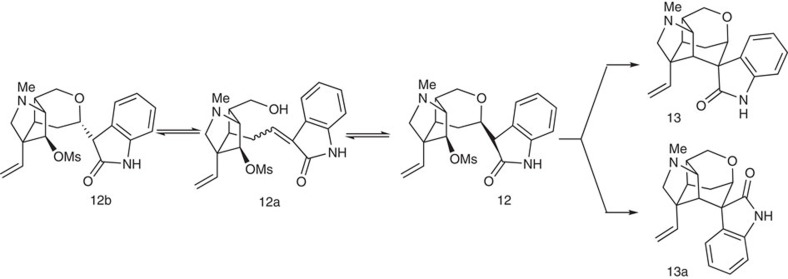
Cyclization of intermediate 9 to form the gelsemine framework. This scheme illustrates the equilibrium between intermediates 12 and 12b via the formation of intermediate 12a. It can be seen that only intermediate 12 can proceed to form the cyclization products 13 and 13a.

**Figure 5:**
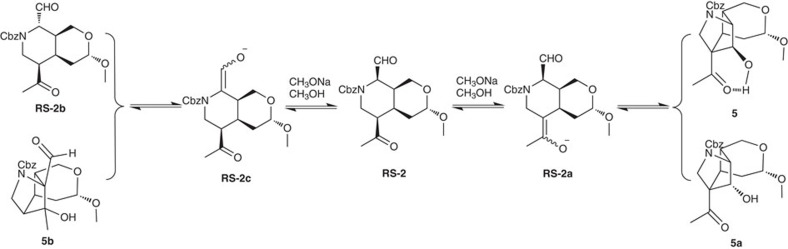
The aldol condensation and possible complications. Enolization of both the aldehyde and the ketone carbonyl groups is possible, while only the cyclization through the ketone carbonyl group enolization can provide the desired product, which is thermodynamically more stable than the other isomer.

Gelsemine may be synthesized from intermediate **RS-1** and oxindole via the condensation of the hemiacetal with oxindole followed by an intramolecular S_N_2 displacement (Fig. 3). Although the condensation may result in four stereoisomers, only two of them may undergo the desired S_N_2 displacement. The other two isomers, however, may either stay intact or undergo an elimination followed by a Michael addition^51^,^52^ to regenerate the four stereoisomers. This equilibrium is shifted to form the desired product after the intramolecular S_N_2 displacement, which is irreversible under the reaction conditions (Figs 4 and 5). The S_N_2 displacement may result in two isomers, one of which is the desired product. Intermediate **RS-1** may be obtained from **RS-2** following a sequence of intramolecular aldol condensation, reduction of the carbonyl group, formation of the sulfonates and then elimination. The intramolecular aldol^53^,^54^ condensation deserves further discussion due to the fact that both the aldehyde and the ketone functionalities may undergo enolization under the reaction conditions, resulting in epimerization of both stereochemical centres attaching the carbonyl groups. Another issue is the direction of the aldol condensation. Since both of the carbonyl groups may be enolized, the aldol condensation from either one may be consequential. However, Cbz is a bulky functional group^55^ and it will play a significant role in preventing the aldehyde from being enolized prior to the ketone enolization. In this case, the potential epimerization of the ketone functionality is irrelevant. The third issue is the stereochemistry of the hydroxyl group even if aldol condensation occurs in the desired direction. This difficulty may be overcome when one realizes that the desired product has a more favourable internal hydrogen bond^56^,^57^ than the other isomer. Finally, formation of **RS-3** and its conversion into **RS-2** is straightforward.

### Synthesis of the (+)-gelsemine

**Figure 6:**
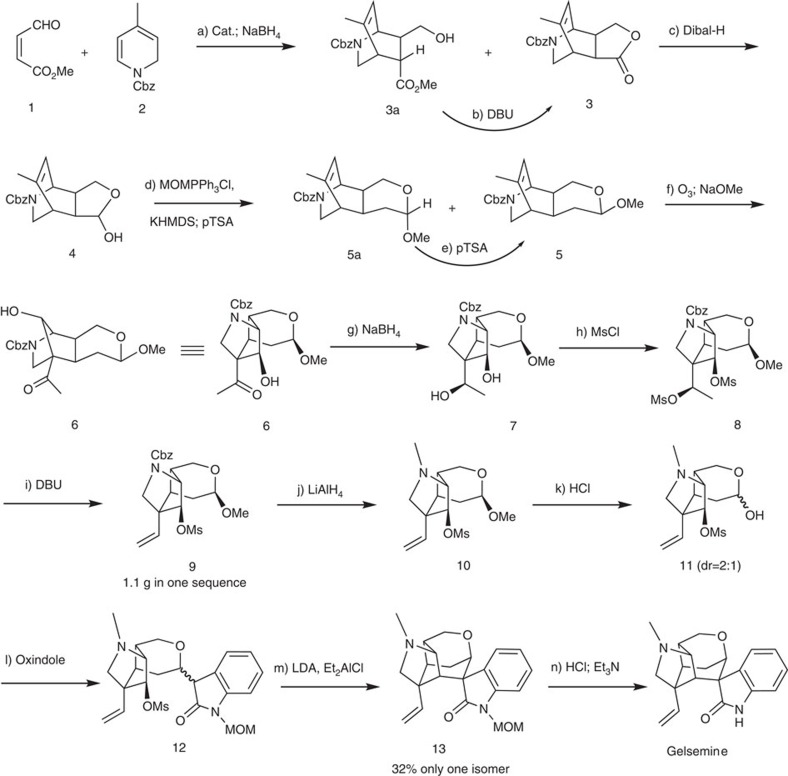
The synthesis of (+)-gelsemine. Reagents and conditions: (**a**) Cat. (0.1 eq), CH_3_CN/H_2_O (20:1), −20 °C, 36 h, then NaBH_4_ (1 eq), 0 °C, 30% for **3a**, 47% for **3**; (**b**) DBU, toluene, reflux, 20 h, 97%; (**c**) Dibal-H (1.05 eq), DCM, −78 °C, 3 h, 90%; (**d**) KHMDS (4.4 eq), MOMPPh_3_Cl (4 eq), THF, 0 °C—rt, 3 h, then **4**, 0 °C, 4 h; *p*TSA (0.1 eq), CH(OMe)_3_, DCM, rt, 93% for **5a** and **5** (**5a**:**5**=1:13); (**e**) pTSA (0.1 eq), CH(OMe)_3_, DCM, rt; (**f**) O_3_, DCM, −78 °C, 30 min; NaOCH_3_ (0.3 eq), CH_3_OH, 0 °C, 24 h, 60%; (**g**) NaBH_4_ (1.1 eq), CH_3_OH, 0 °C, 30 min, 93%; (**h**) MsCl (3 eq), DMAP (3 eq), Et_3_N (5 eq), DCM, 0 °C, quantitative; (**i**) DBU, toluene, reflux, 24 h, 85%; (**j**) LiAlH_4_ (1.2 eq), THF, 0 °C, 10 h, 86%; (**k**) 6 M HCl, THF, H_2_O, 3 h, 96%; (**l**) piperidine, 1-MOM-oxindole (1.5 eq), CH_3_OH, reflux, 86%; (**m**) LDA (1.2 eq), Et_2_AlCl (5 eq), toluene, 32%; (**n**) 6 M HCl, THF, 50 °C, 24 h; Et_3_N, CH_3_OH, 55 °C, 24 h, 70%. DBU, 1,8-diazabicycloundec-7-ene; Dibal-H, diisobutyl aluminium hydride; KHMDS, potassium hexamethyldisilazane; *p*TSA, *p*-toluenesulfonic acid; DCM, dichloromethane; MsCl, methanesulfonyl chloride; DMAP, 4-dimethylaminopyridine; LDA, lithium diisopropylamide; rt, room temperature.

On the basis of the above analysis, the synthetic strategy seemed feasible. If intermediate **3** is made asymmetric, then gelsemine will be made asymmetric. Thus, after a brief literature search^58^,^59^, an asymmetric Diels–Alder reaction was designed and the synthesis began with dihydropyridine **1** (Fig. 6), which may be prepared from 4-methylpyridine in large scale^60^.

**Figure 7:**
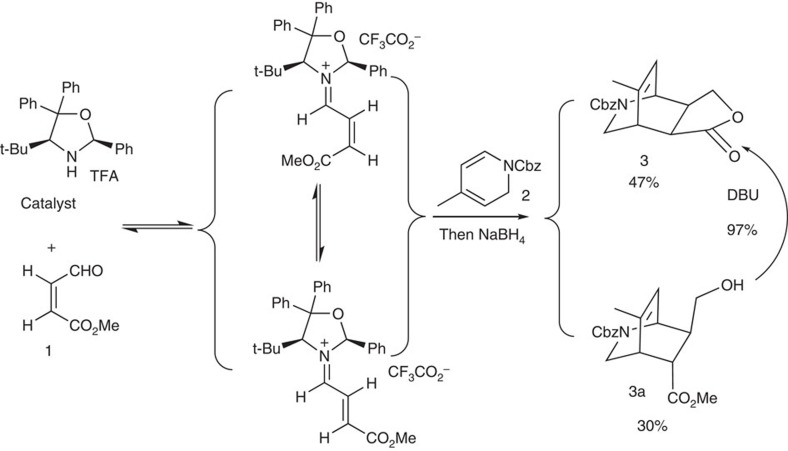
Intermediates leading to the formation of 3 and 3a. The reaction consequence indicates that the carbon–carbon double-bond isomerization of the iminium salt occurred at a rate comparable to that of the cycloaddition.

**Figure 8:**
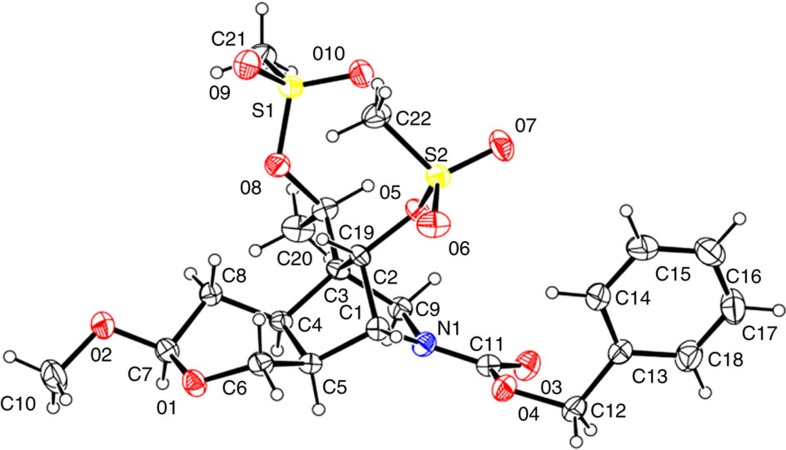
The X-ray crystallographic structure of intermediate 8. ORTEPs are included in the Supporting Information as a separate file. CCDC 1056043 contains the supplementary crystallographic data.

Gratifyingly, the yield of the desired endo product was 47% after reduction of the aldehyde carbonyl group with sodium borohydride, and its enantio excess was determined using chiral high-performance liquid chromatography (HPLC) to be 99.7%, while the exo product was not detected. It was surprising that intermediate **3a** was also produced in 30% yield. Since intermediate **3** was stable under the reaction conditions, **3a** may be a result of the double-bond isomerization of the enal during the catalytic process^61^, and the rate of the double-bond isomerization was comparable to that of the Diels–Alder cycloaddition (Fig. 7). Fortunately, **3a** was converted into **3** with DBU (1,8-diazabicycloundec-7-ene) in refluxing toluene in 97% yield, which brought the total yield of the Diels–Alder cycloaddition to 76%. Intermediate **3** was then further selectively reduced to the hemiacetal **4** using Dibal-H at −78 °C in 94% yield. The subsequent Wittig reaction furnished the methyl enol ether, which was directly treated with trimethyl orthoformate and a catalytic amount of *p*-toluenesulfonic acid to provide intermediate **5** and **5a** (13:1) as a separable mixture in 93% combined yield. Although **5a** may be used as well, it was converted into **5** by treating it with *p*TSOH in methylene chloride (DCM) and only **5** was used for the next step. After a conventional ozonolysis of intermediate **5** in DCM, the resulting dicarbonyl intermediate was directly treated with sodium methoxide in methanol at 0 °C due to the fact that the dicarbonyl intermediate was unstable for storage. To our delight, the aldol reaction afforded the desired product **6** in 60% combined yield. However, the reaction of **6** with the methanesulfonyl chloride resulted in a complex mixture. Thus, the hydroxyketone intermediate **6** was reduced to diol **7** with sodium borohydride (97%) and the formation of disulfonate **8** with methanesulfonyl chloride was quantitative, the structure of which was confirmed through X-ray crystallographic analysis (Fig. 8). Treatment of intermediate **8** with DBU (1,8-diazabicycloundec-7-ene) in refluxing toluene led to the formation of alkene **9** (85%) and reduction of the Cbz protective group to methyl with lithium aluminium hydride in THF afforded **10** in 86% yield. Subsequent acid hydrolysis of the acetal with aqueous hydrochloric acid in THF provided hemiacetal **11** (96%).

With the key intermediate in hand, we began to test the condensation of **11** with methoxymethyl oxindole and the subsequent S_N_2 displacement, another key reaction for the synthesis of gelsemine. As expected, the condensation of intermediate **11** with oxindole in refluxing methanol and a catalytic amount of piperidine afforded the desired product **12** (85%) as an inseparable mixture of all four possible isomers. The seemed straightforward intramolecular S_N_2 substitution reaction turned out to be problematic. Many reaction conditions were tested (NaH/THF; NaOCH_3_/CH_3_OH; KO^*t*^Bu/THF; KO^*t*^Bu/THF/Bu^*t*^OH; LDA/THF; CsF/DMF^62^; LiHMDS/THF; LiHMDS/HMPA/THF; LiHMDS/LiCl/THF, LiHMDS/ZnCl_2_/THF; LiHMDS/DMSO; LDA/Et_2_AlCl/THF; LiHMDS/Me_2_AlCl/toluene; LiHMDS/Me_2_AlCl/THF; NaHMDS/Me_2_AlCl/THF, NaH/DMF) but all turned into a complex product mixture. However, when intermediate **12** was treated with LDA and then diethylaluminum chloride in toluene at 90 °C, the reaction furnished the desired product in 32% yield as a single isomer. Finally, acid hydrolysis of the methyl group from the methoxymethyl protective group and removal of the resulting hydroxymethyl with triethylamine converted **13** into (+)-gelsemine in 70% combined yield. The synthetic material is identical to the natural product in terms of carbon and proton NMR spectra and optical rotation (see Supplementary Fig. 15).

## Discussion

The total synthesis of (+)-gelsemine is completed in a highly enantioselective manner from readily accessible starting materials. This synthesis features an enantioselective organocatalytic Diels–Alder reaction, a formidable intramolecular aldol cyclization and a challenging intramolecular S_N_2 displacement. The combination of all these features resulted in exceptional overall synthetic efficiency: the enantio excess is over 99%, and the total yield is about 5%.

## Methods

### General

All reagents were reagent grade and used without purification, unless otherwise noted. All reactions involving air- or moisture-sensitive reagents or intermediates were performed under an inert atmosphere of argon in glassware that was oven dried. Reaction temperatures referred to the temperature of the cooling/heating bath. Chromatography was performed using forced flow (flash chromatography) of the indicated solvent system on 230-400 mesh silica gel (Silicycle flash F60), unless otherwise noted. ^1^H NMR and ^13^C NMR spectra were recorded on a Bruker AV-400 or 500 MHz spectrometer. Chemical shifts were referenced to the deuterated solvent (for example, for CDCl_3_, *δ*=7.27 p.p.m. and 77.0 p.p.m. for ^1^H and ^13^C NMR, respectively) and reported in parts per million (p.p.m., *δ*) relative to tetramethylsilane (*δ*=0.00 p.p.m.). Coupling constants (*J*) were reported in Hz and the splitting abbreviations used were: s, singlet; d, doublet; t, triplet; q, quartet; m, multiplet; comp, overlapping multiplets of magnetically non-equivalent protons; br, broad; app, apparent. Reactions were monitored using thin-layer chromatography carried out on 0.25-mm E. Merck silica gel plates (60F-254) using ultraviolet light as the visualizing agent or an ethanolic solution of phosphomolybdic acid, cerium sulfate and heat as developing agents. Optical rotations were measured on a PerkinElmer 341 polarimeter. Enantiomeric ratios were determined by chiral HPLC using a chiralpak AD-H (amylose tris(3,5-dimethylphenylcarbamate) coated on 5-μm silica gel) with hexane and i-PrOH as eluents. Tetrahydrofuran, benzene, toluene and diethyl ether were distilled from Na and diphenylketone. DCM, *N*,*N*-diisopropylethylamine and triethylamine were distilled from calcium hydride, while methanol was distilled from dry magnesium turnings immediately before use.

For ^1^H and ^13^C NMR spectra of compounds, see Supplementary Figs 1–14. For the comparisons of ^1^H spectra of the natural and synthetic gelsemine, see Supplementary Fig. 15. For the HPLC of **3**, see Supplementary Fig. 16. For the experimental procedures and spectroscopic and physical data of compounds and the crystallographic data of compound **8**, see Supplementary Methods.

## Additional information

**Accession codes:** The X-ray crystallographic coordinates for structures **8** reported in this study have been deposited at the Cambridge Crystallographic Data Centre (CCDC), under deposition number 1056043. These data can be obtained free of charge from The Cambridge Crystallographic Data Centre via www.ccdc.cam.ac.uk/data_request/cif.

**How to cite this article:** Chen, X. *et al*. Total synthesis of (+)-gelsemine via an organocatalytic Diels–Alder approach. *Nat. Commun.* 6:7204 doi: 10.1038/ncomms8204 (2014).

## Supplementary Material

Supplementary Information Supplementary Figures 1-16, Supplementary Methods and Supplementary References

## Figures and Tables

**Figure 1:**
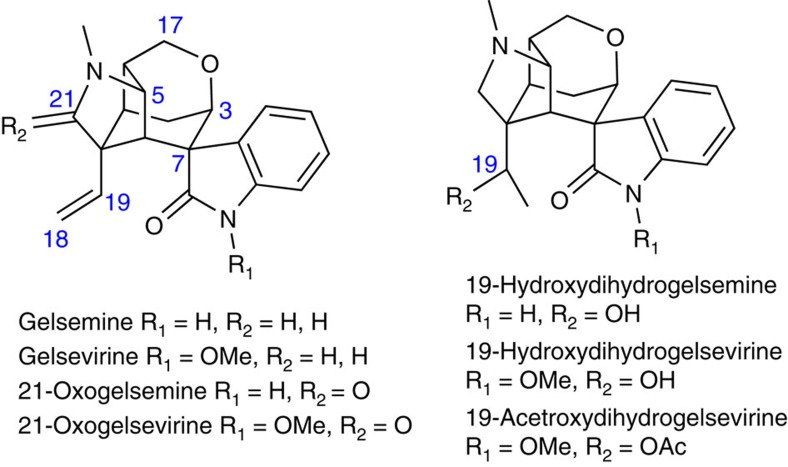
The structures of gelsemium alkaloids. The difference between the members of the gelsemium alkaloids is the presence of the functional groups in the unique carbon skeleton. The major difference appeared in C-19 and C-21.

**Figure 2:**
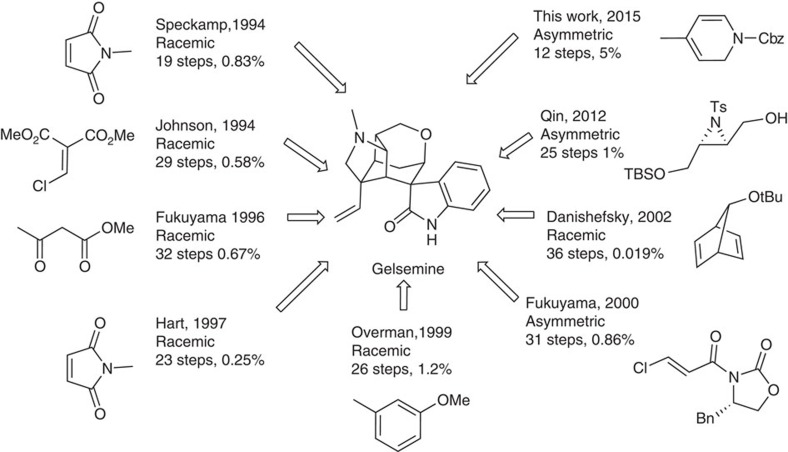
Schematic summary of the previous total syntheses of gelsemine. Among the seven total syntheses completed so far, two of them were asymmetric and the overall yields were around 1%. This molecule has been an active target of total synthesis during the past two decades.
