# Supplementary material for: Total synthesis of (+)-gelsemine via an organocatalytic Diels–Alder approach
Source: Nat Commun. 2015 May 21;6:7204. doi: 10.1038/ncomms8204 (PMC4647982; doi:10.1038/ncomms8204)
Supplement: Supplementary Information — Supplementary Figures 1-16, Supplementary Methods and Supplementary References [file ncomms8204-s1.pdf]

Supplementary figures

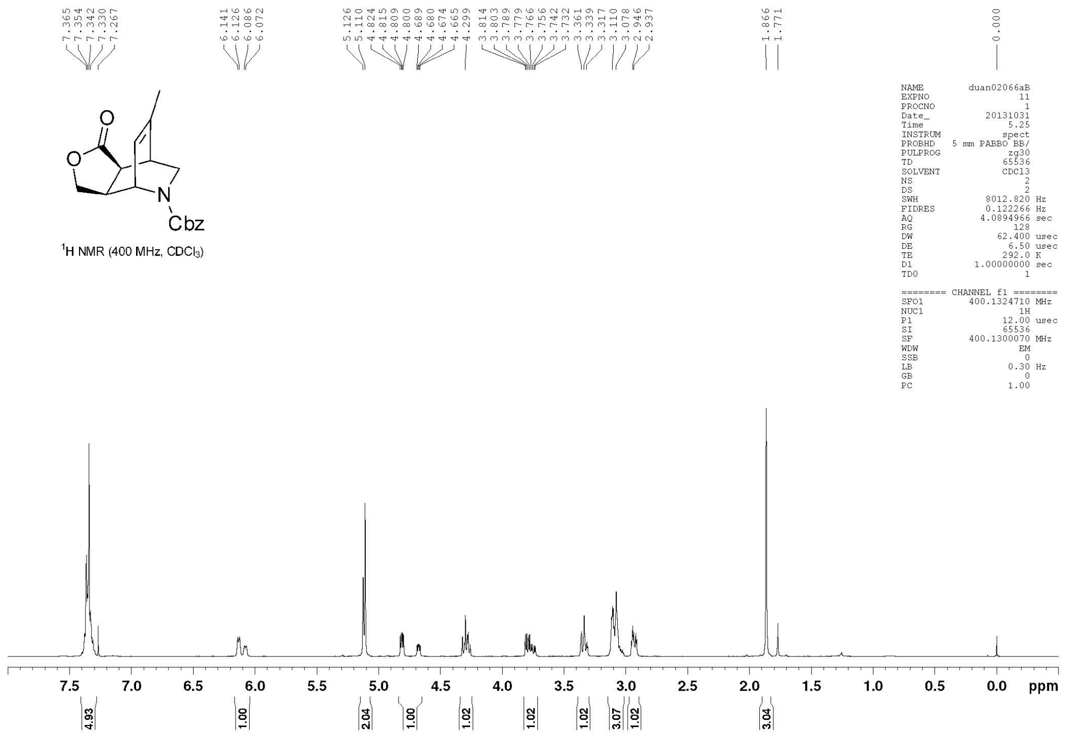

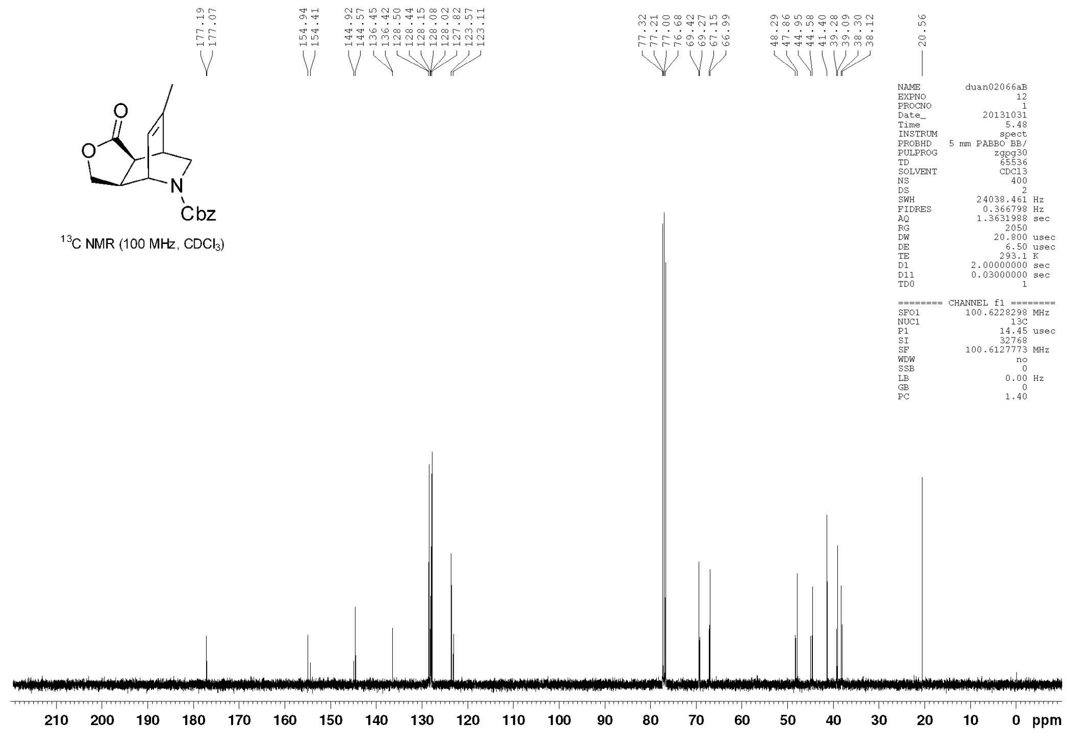

Supplementary figure 1. ^{1}H and ^{13}C NMR spectra for substrate **3**

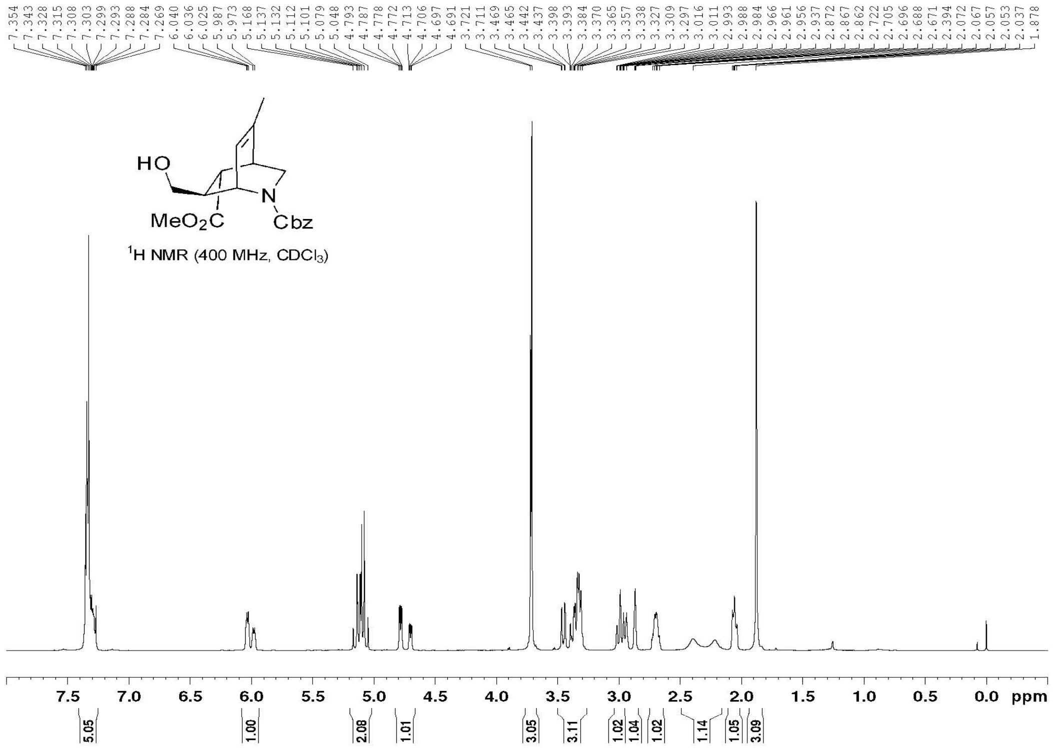

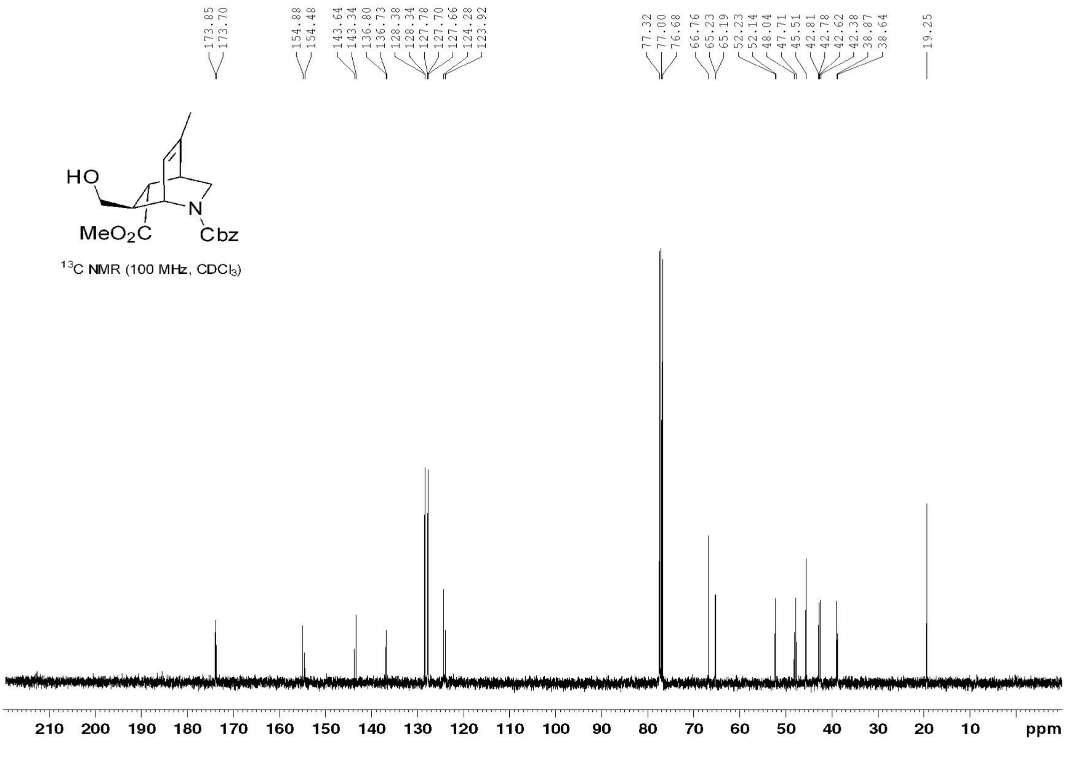

Supplementary figure 2. ^{1}H and ^{13}C NMR spectra for substrate **3a**

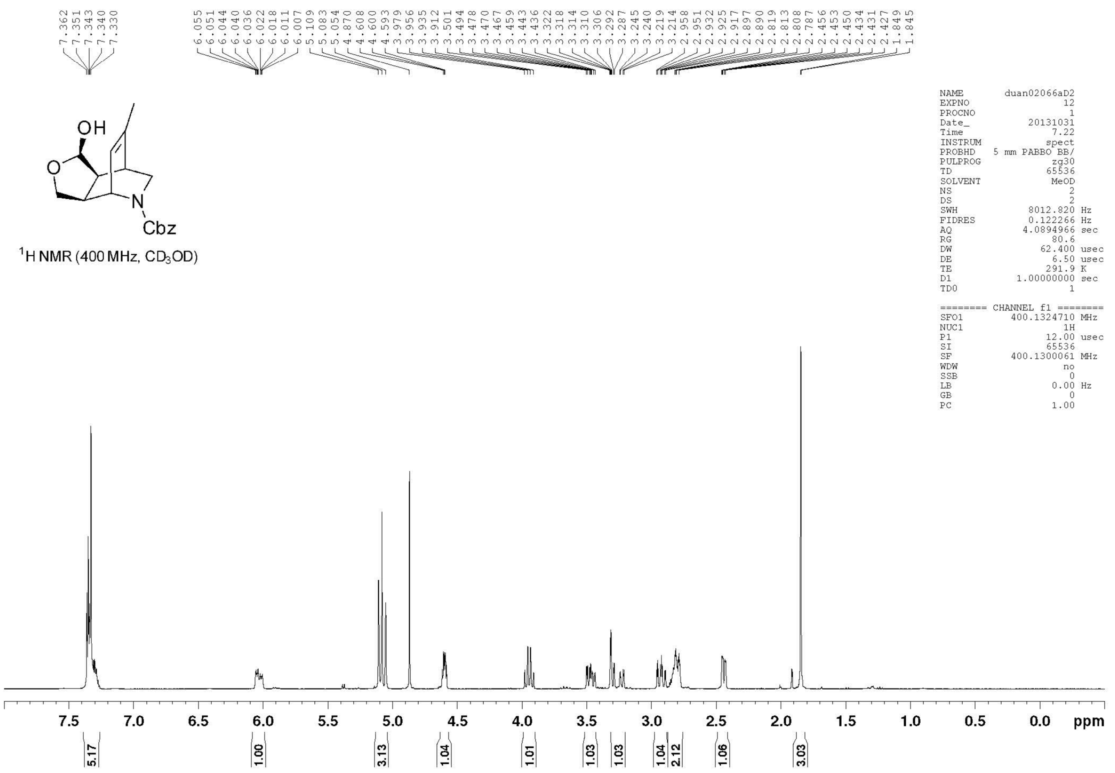

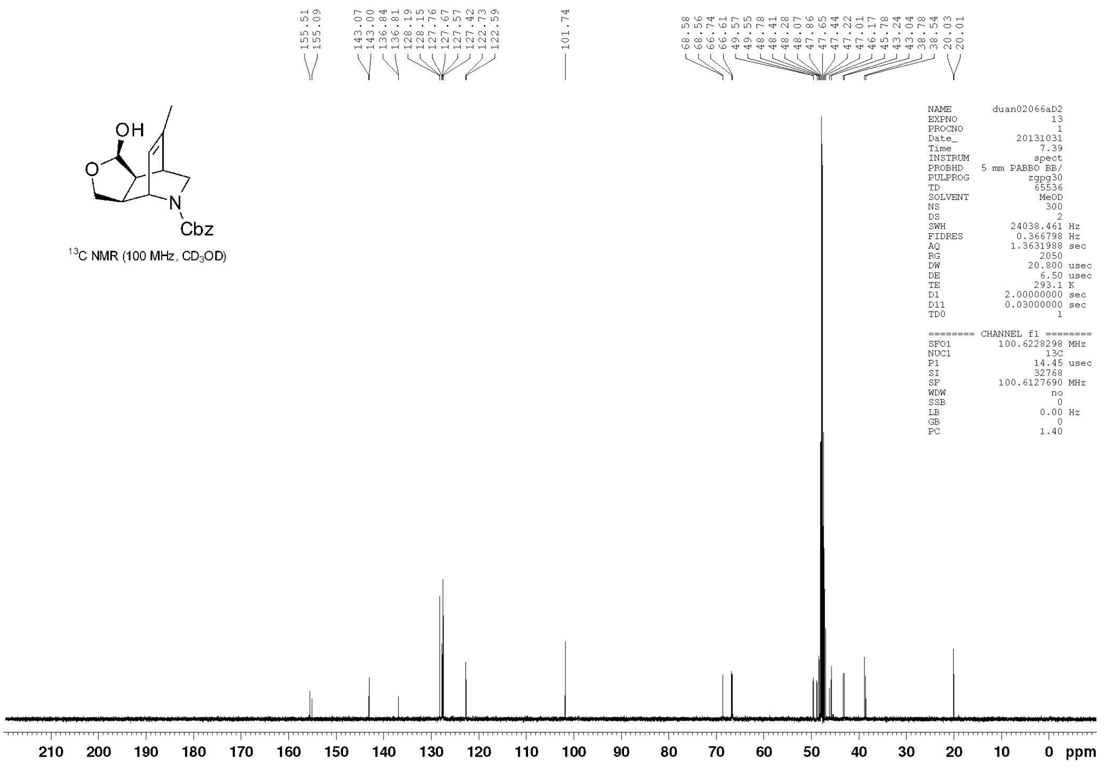

Supplementary figure 3. ^{1}H and ^{13}C NMR spectra for substrate **4**

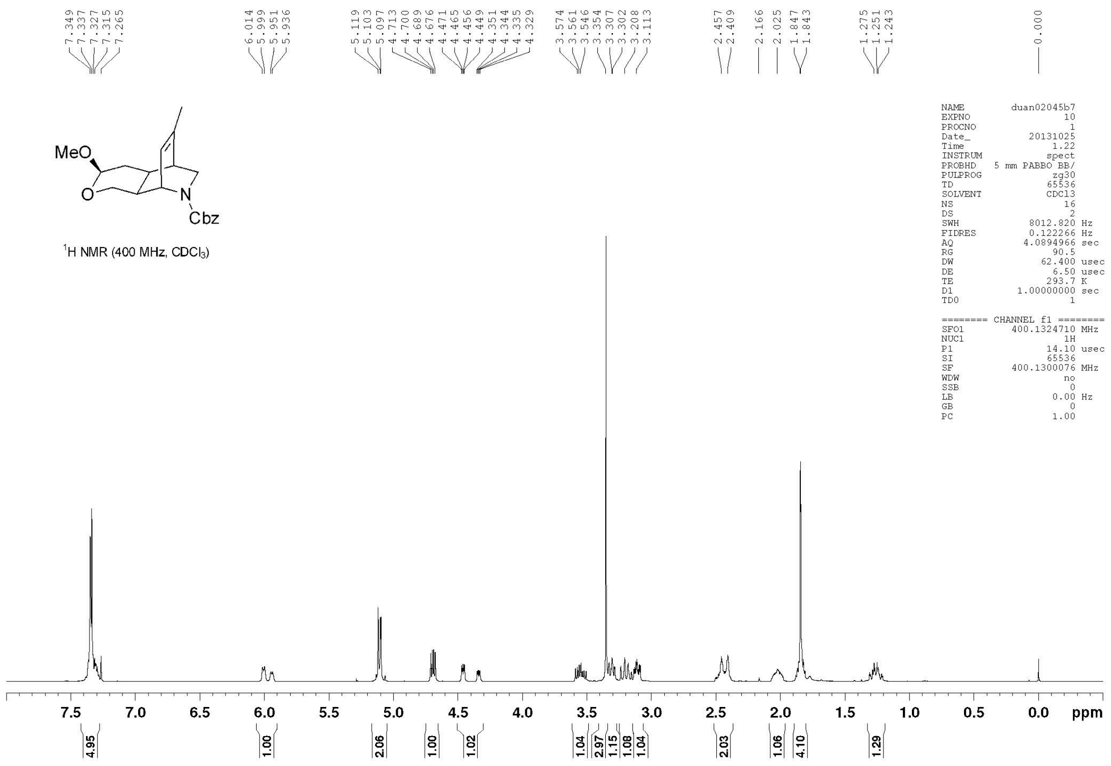

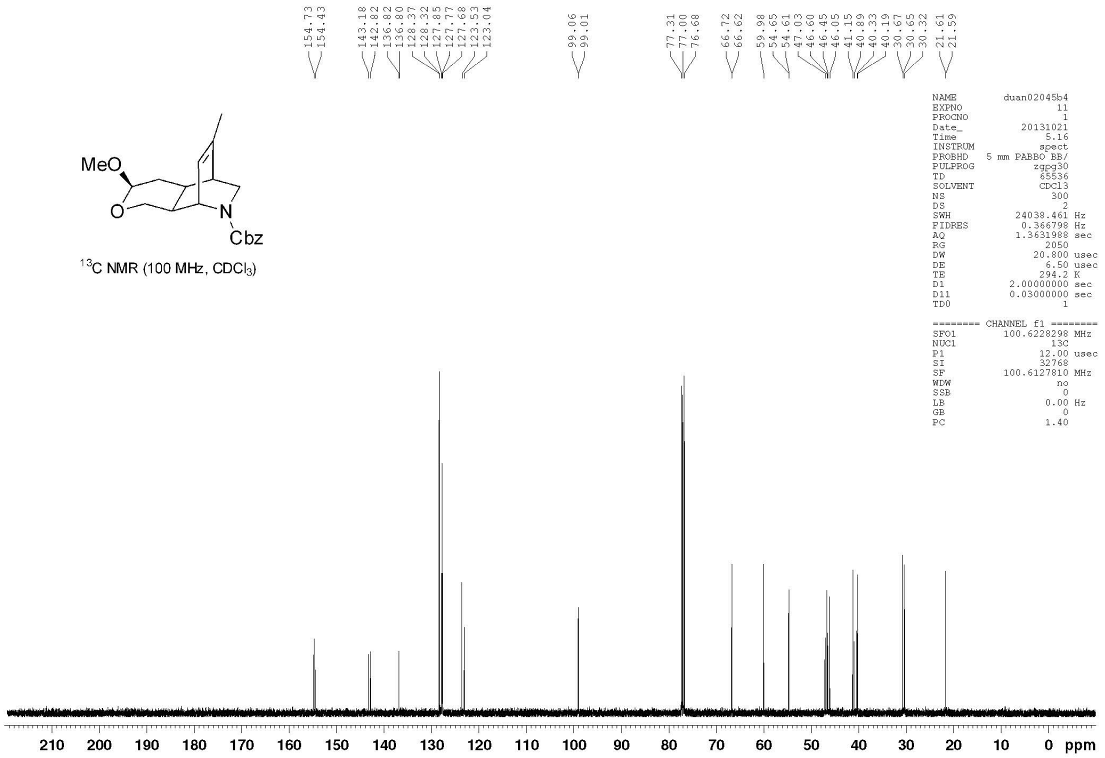

Supplementary figure 4. ^{1}H and ^{13}C NMR spectra for substrate **5**

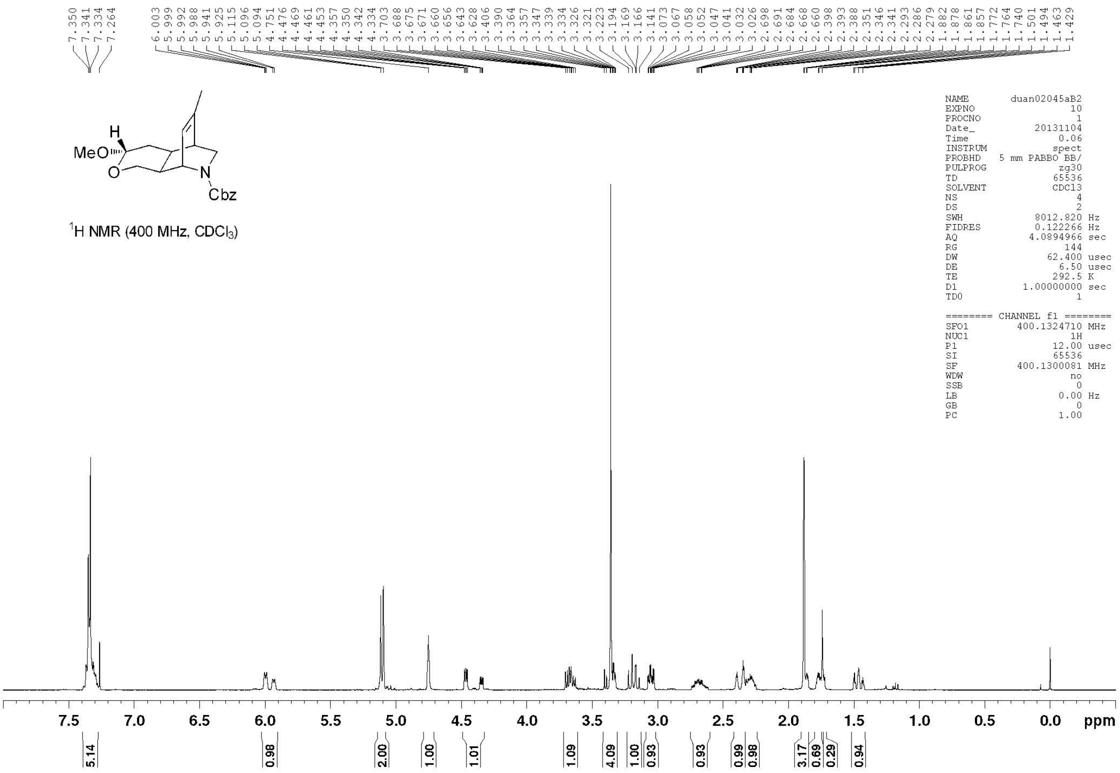

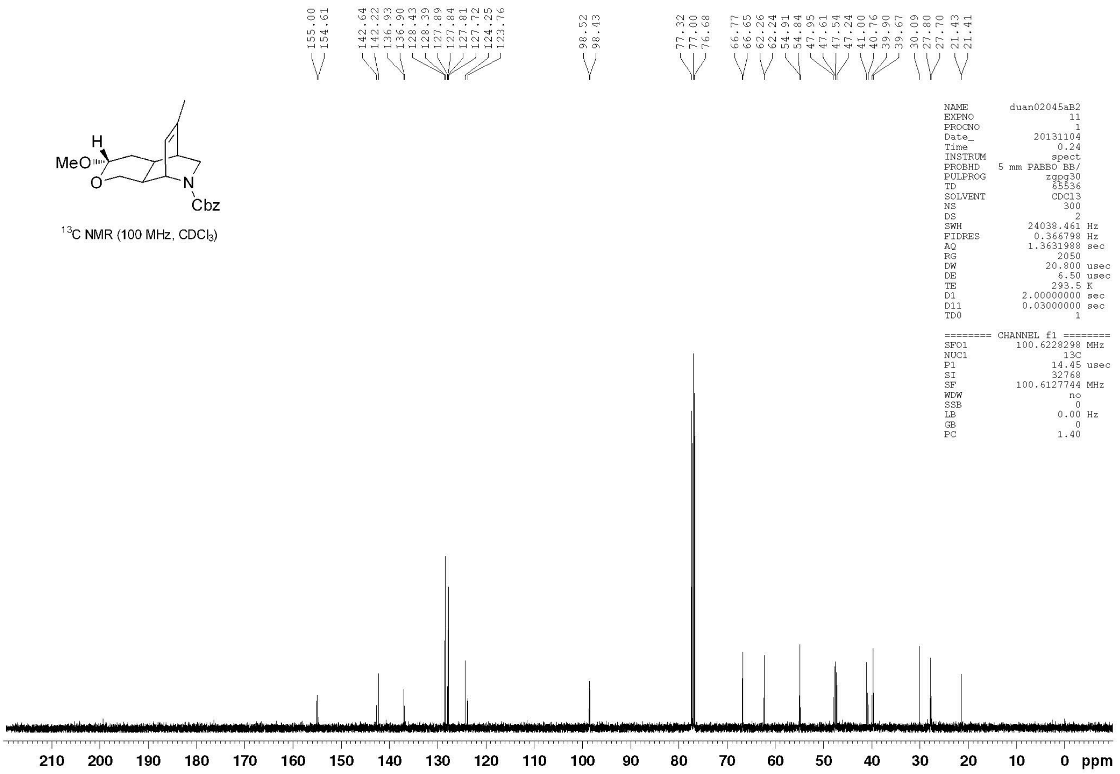

Supplementary figure 5. ^{1}H and ^{13}C NMR spectra for substrate **5a**

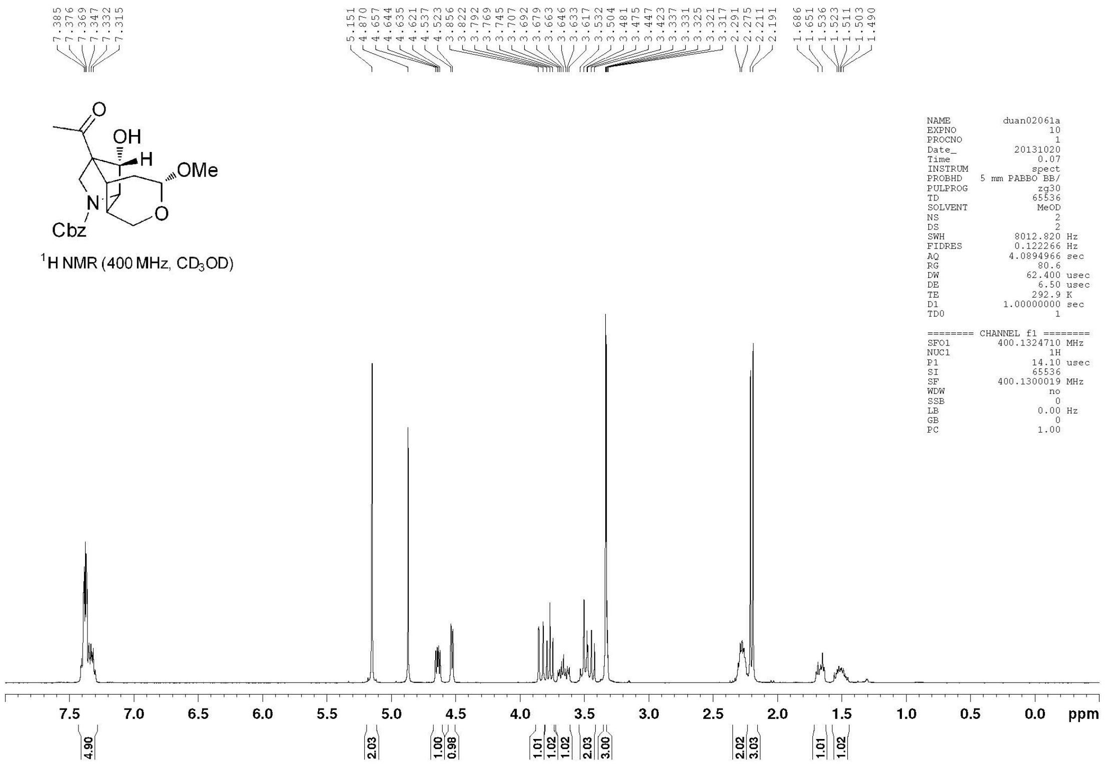

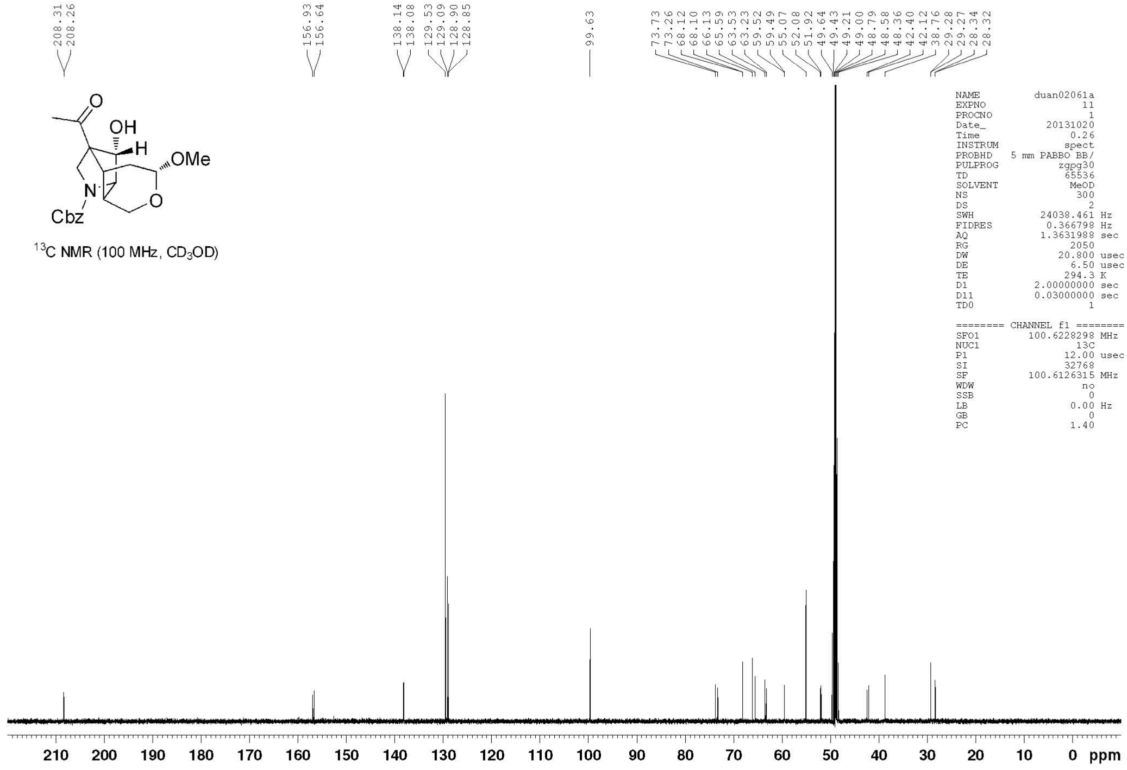

Supplementary figure 6. ^{1}H and ^{13}C NMR spectra for substrate **6**

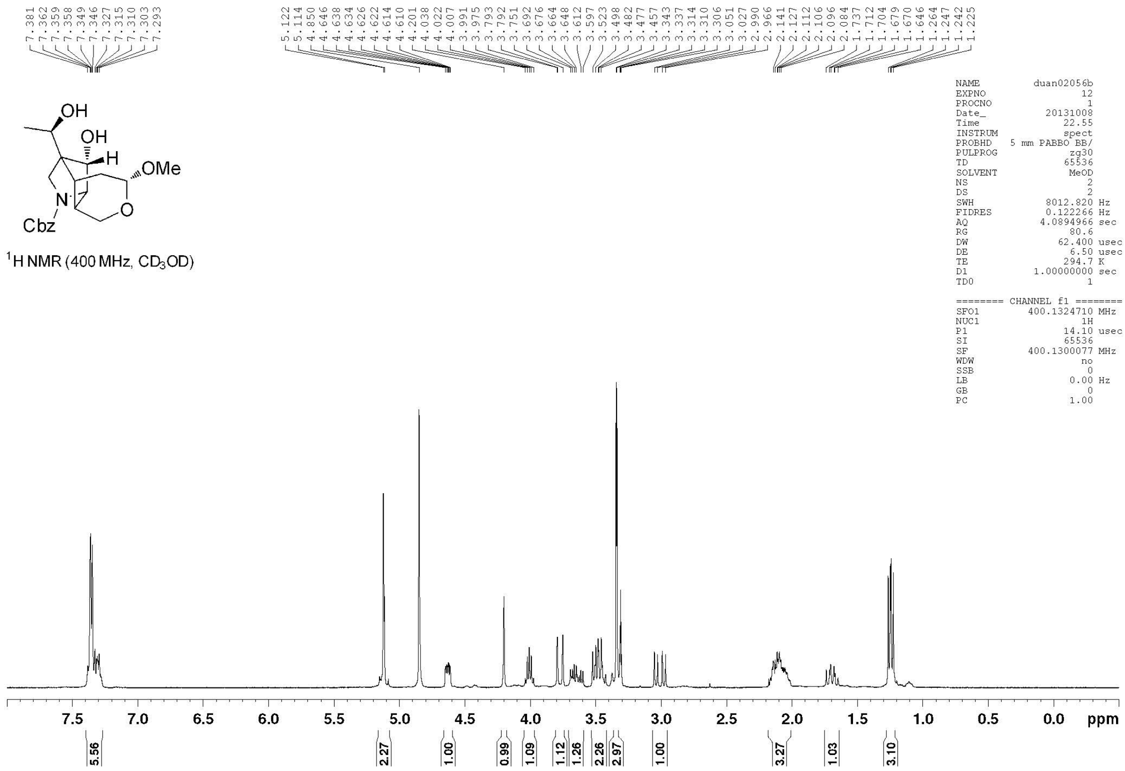

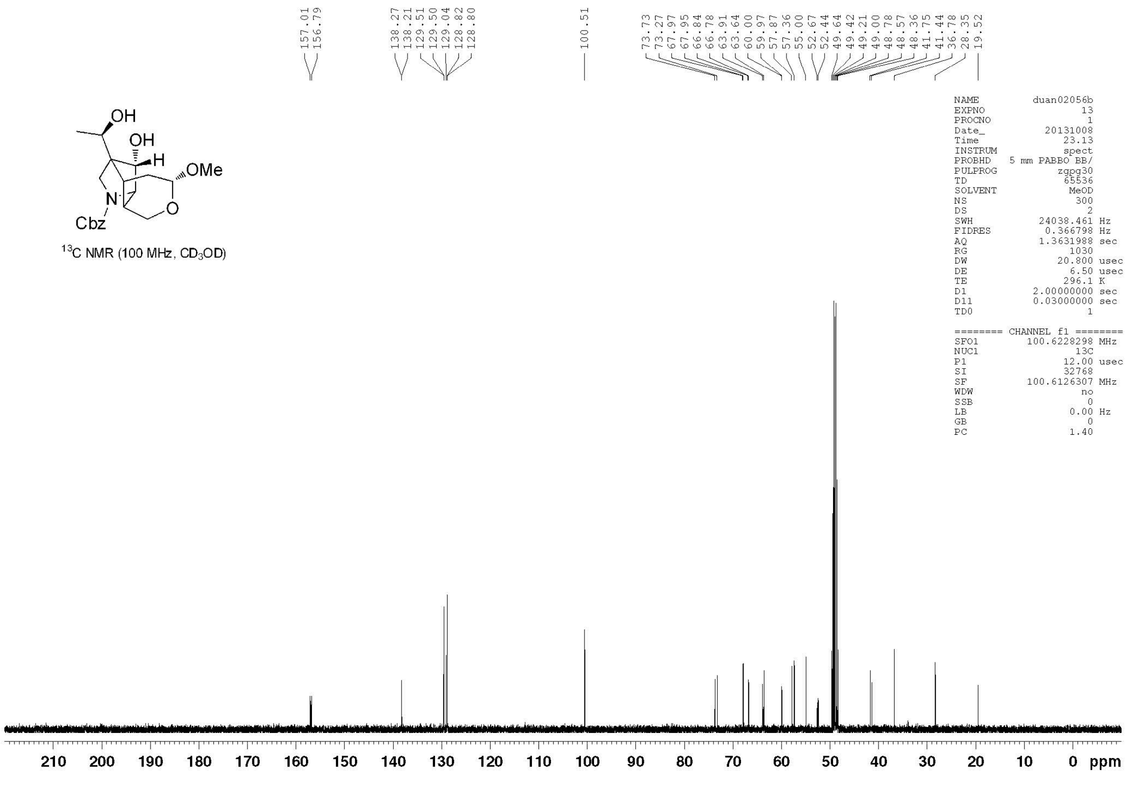

Supplementary figure 7. ^{1}H and ^{13}C NMR spectra for substrate **7**

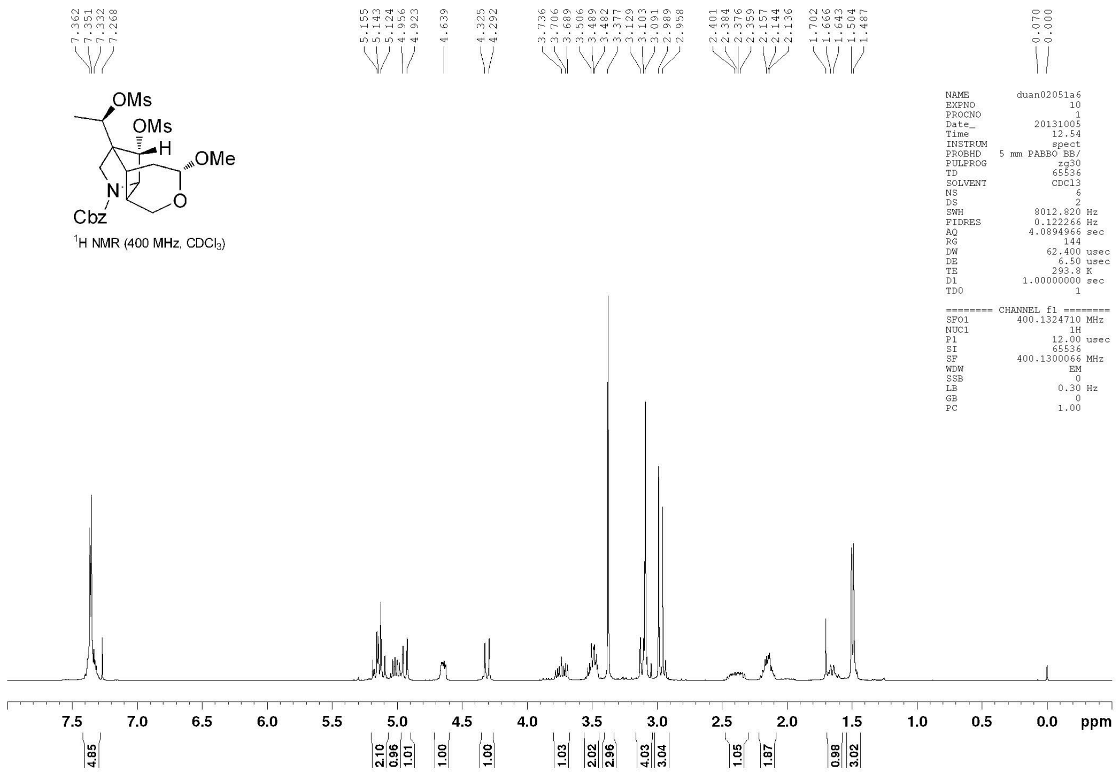

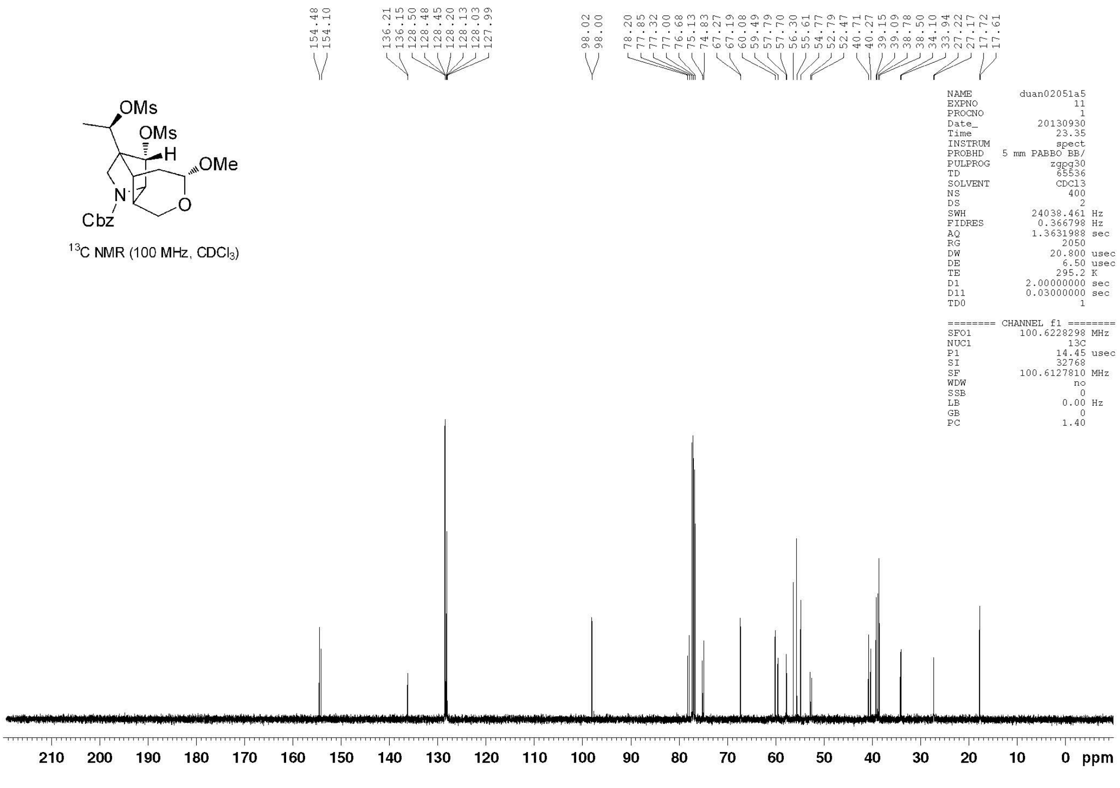

Supplementary figure 8. ^{1}H and ^{13}C NMR spectra for substrate **8**

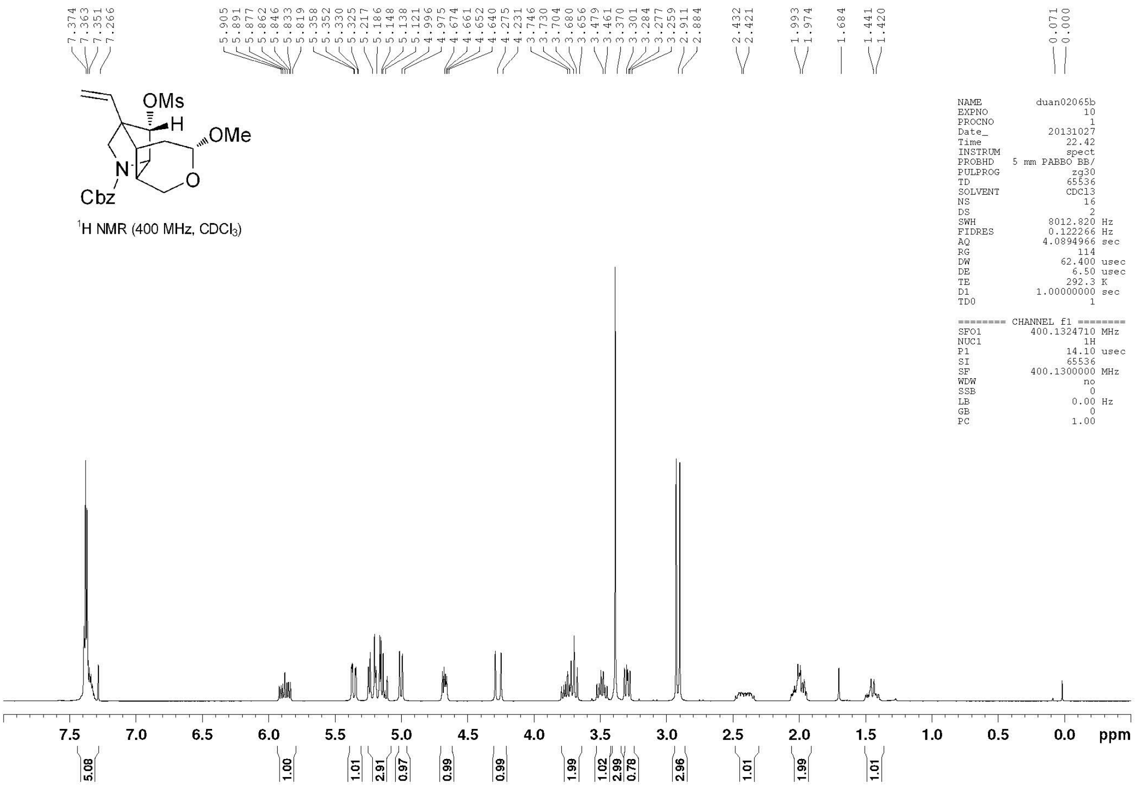

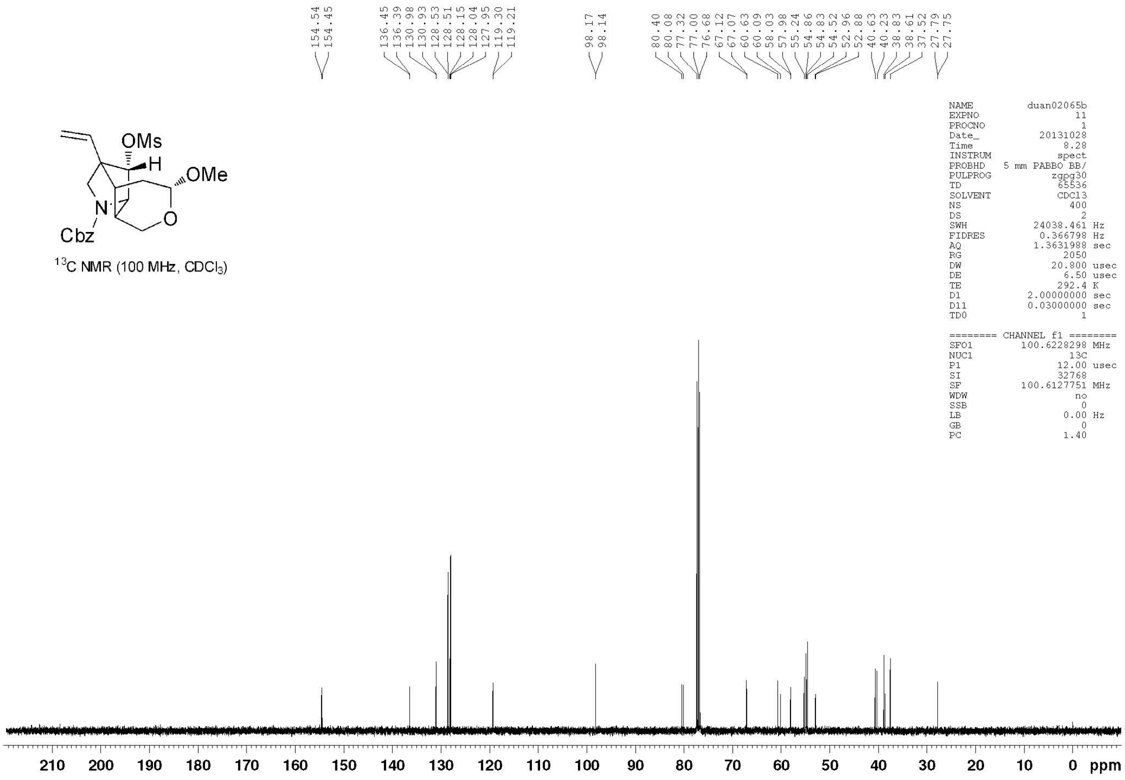

Supplementary figure 9. ^{1}H and ^{13}C NMR spectra for substrate **9**

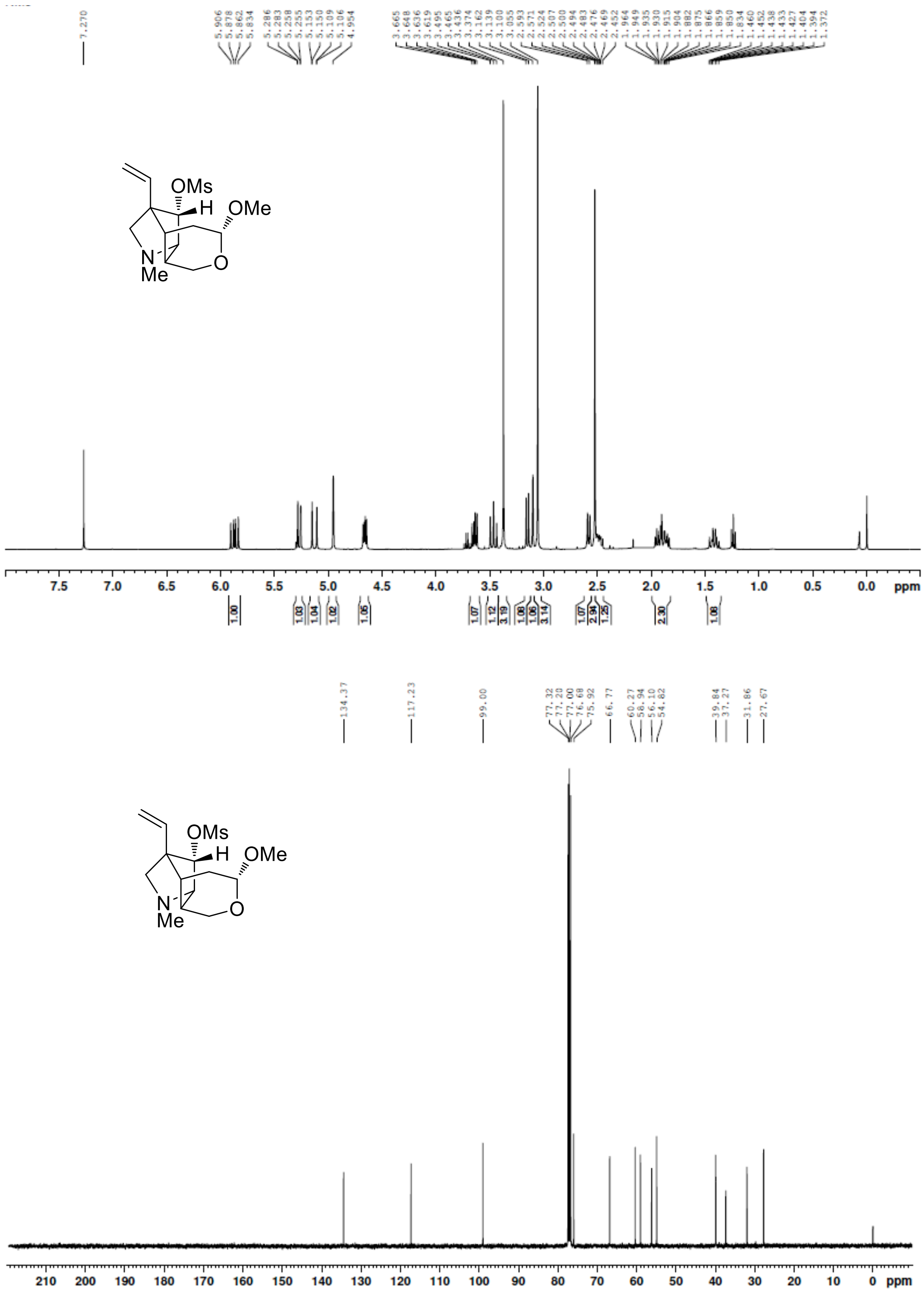

Supplementary figure 10. ^{1}H and ^{13}C NMR spectra for substrate **10**

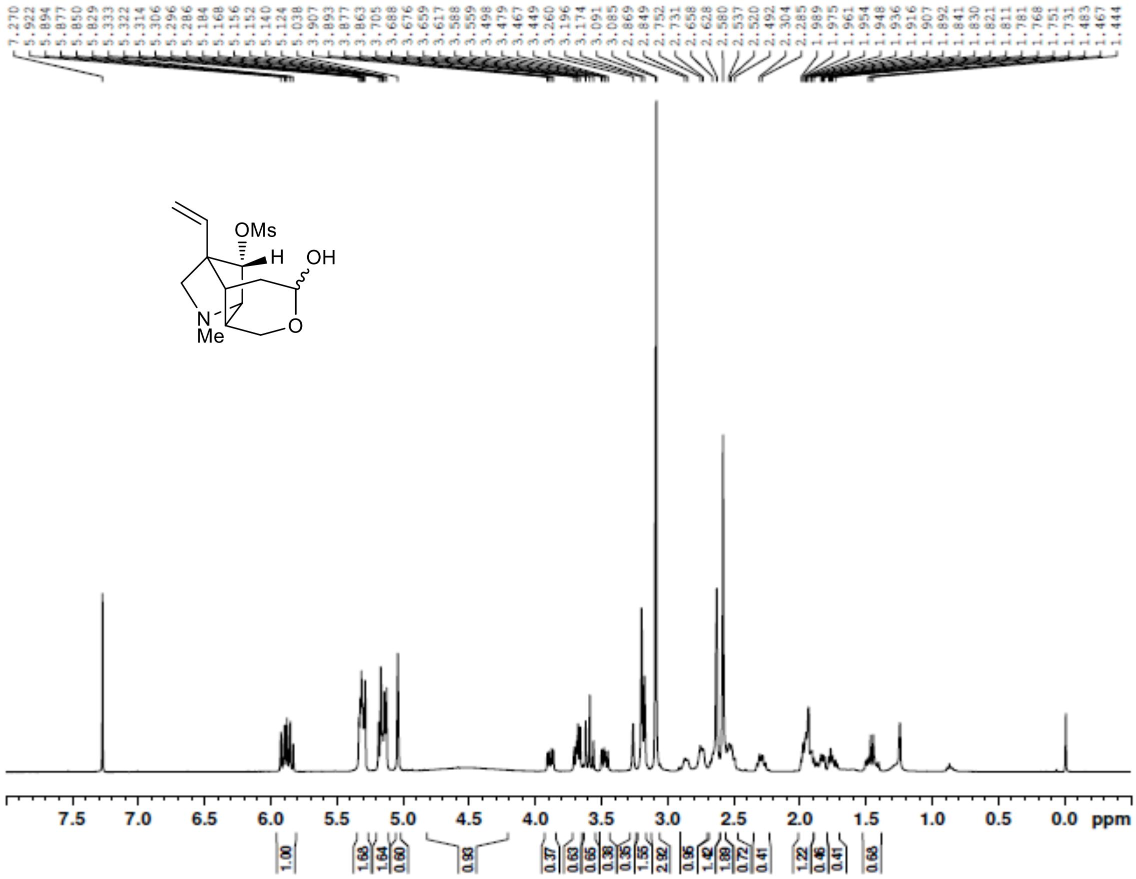

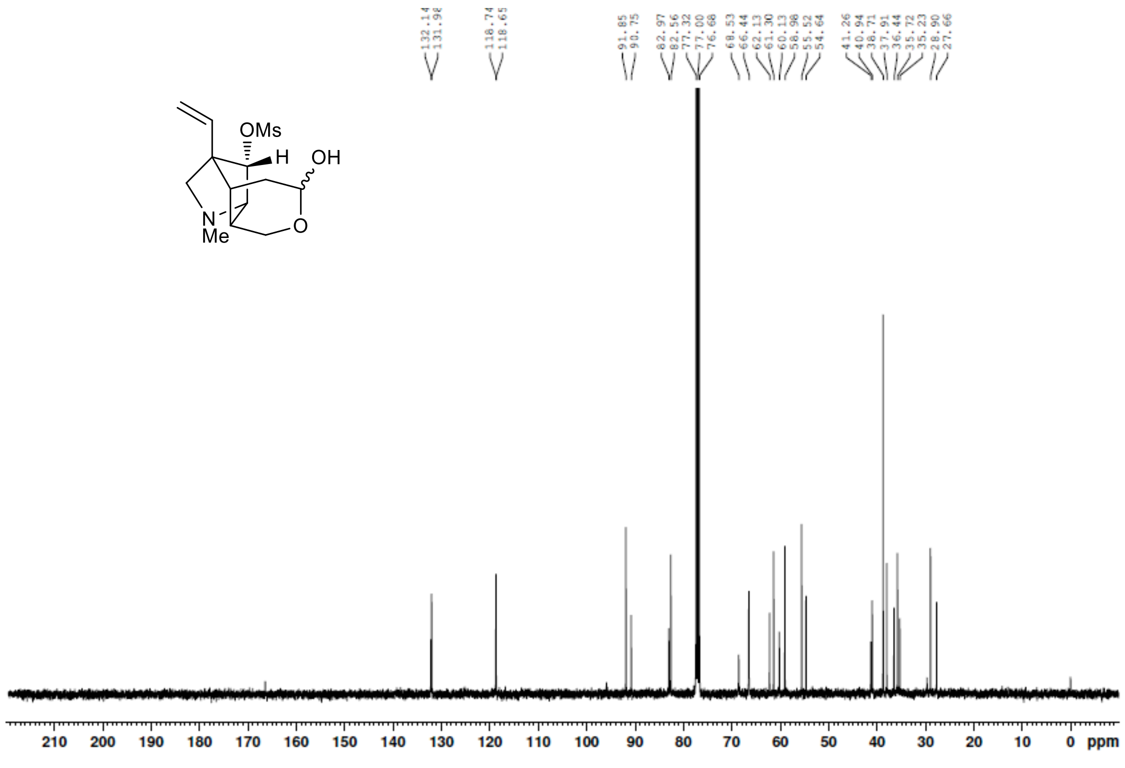

Supplementary figure 11. ^{1}H and ^{13}C NMR spectra for substrate **11**

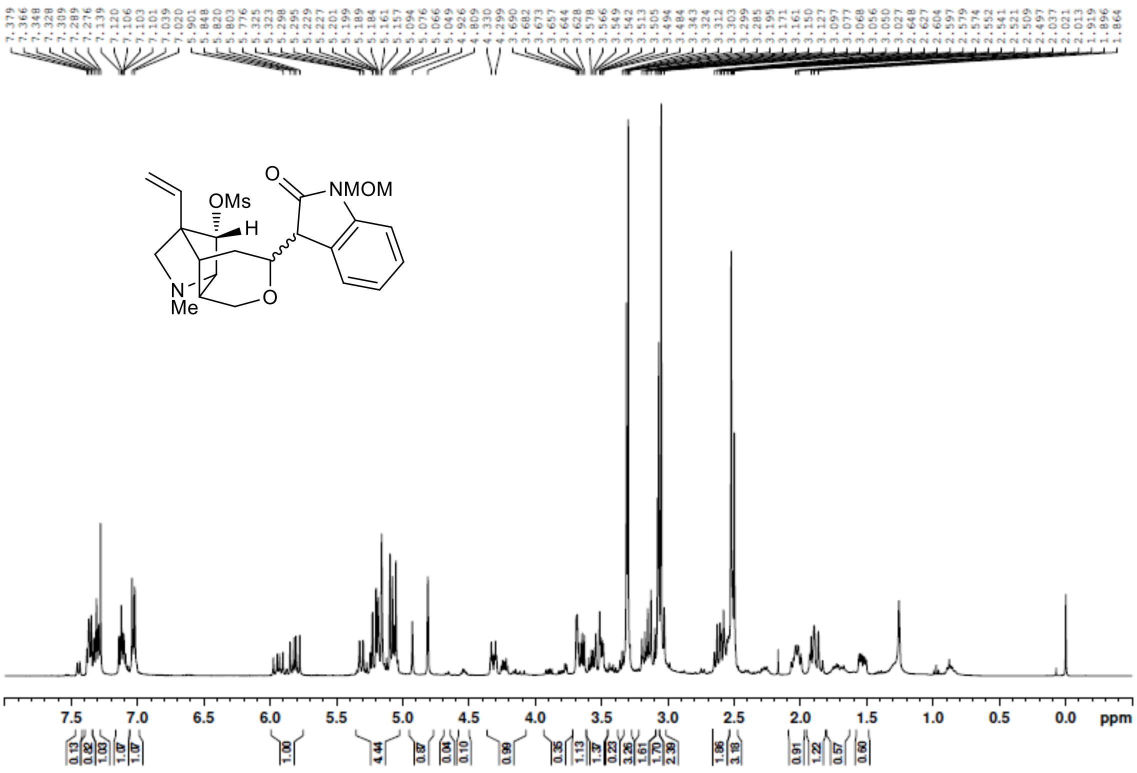

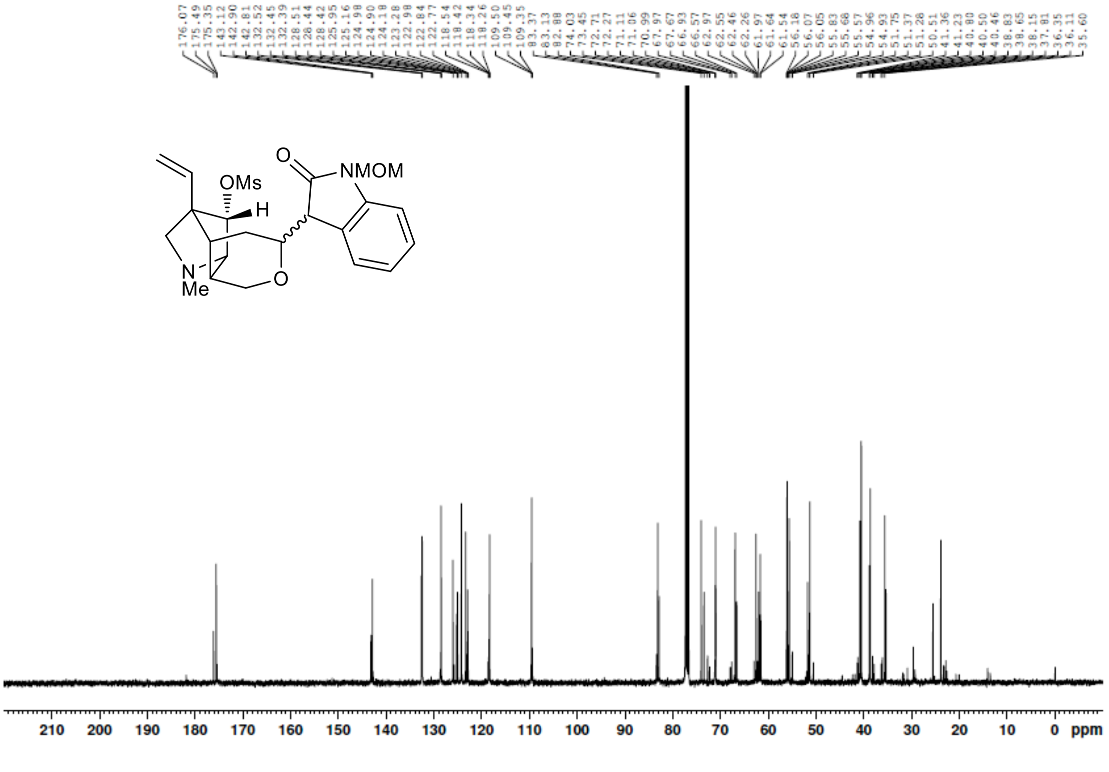

Supplementary figure 12. ^{1}H and ^{13}C NMR spectra for substrate **12**

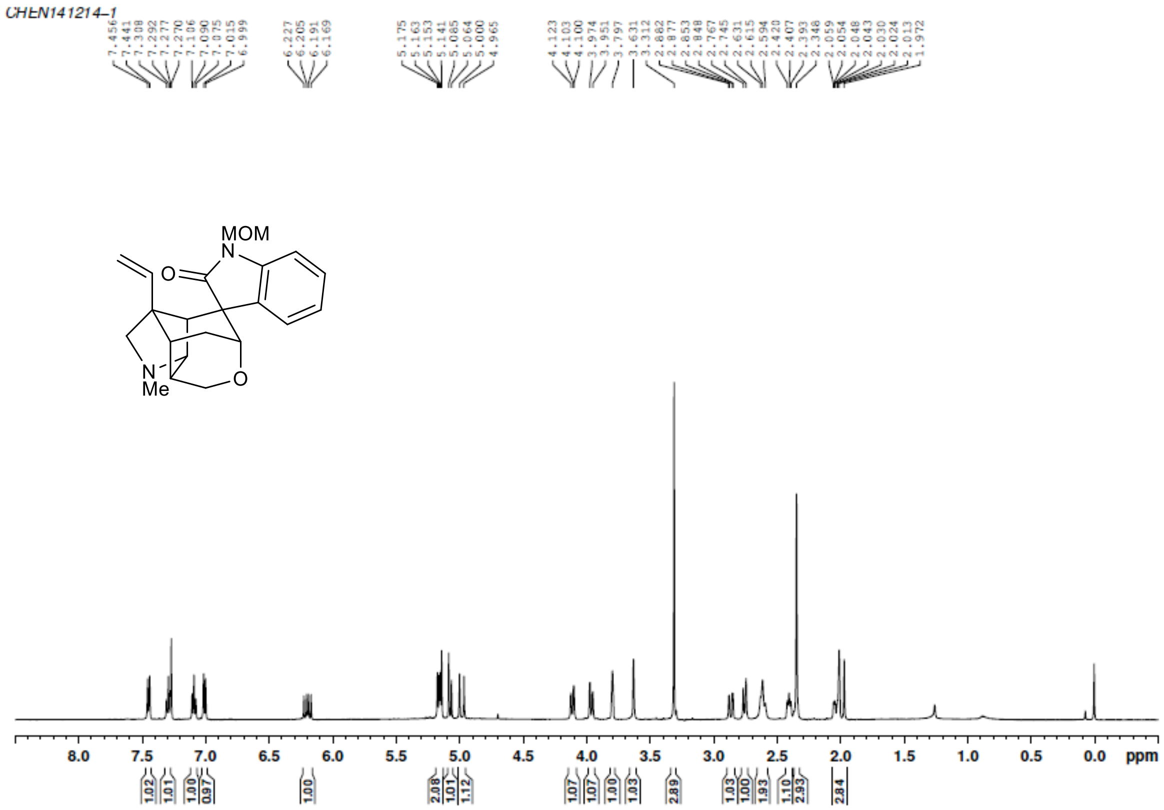

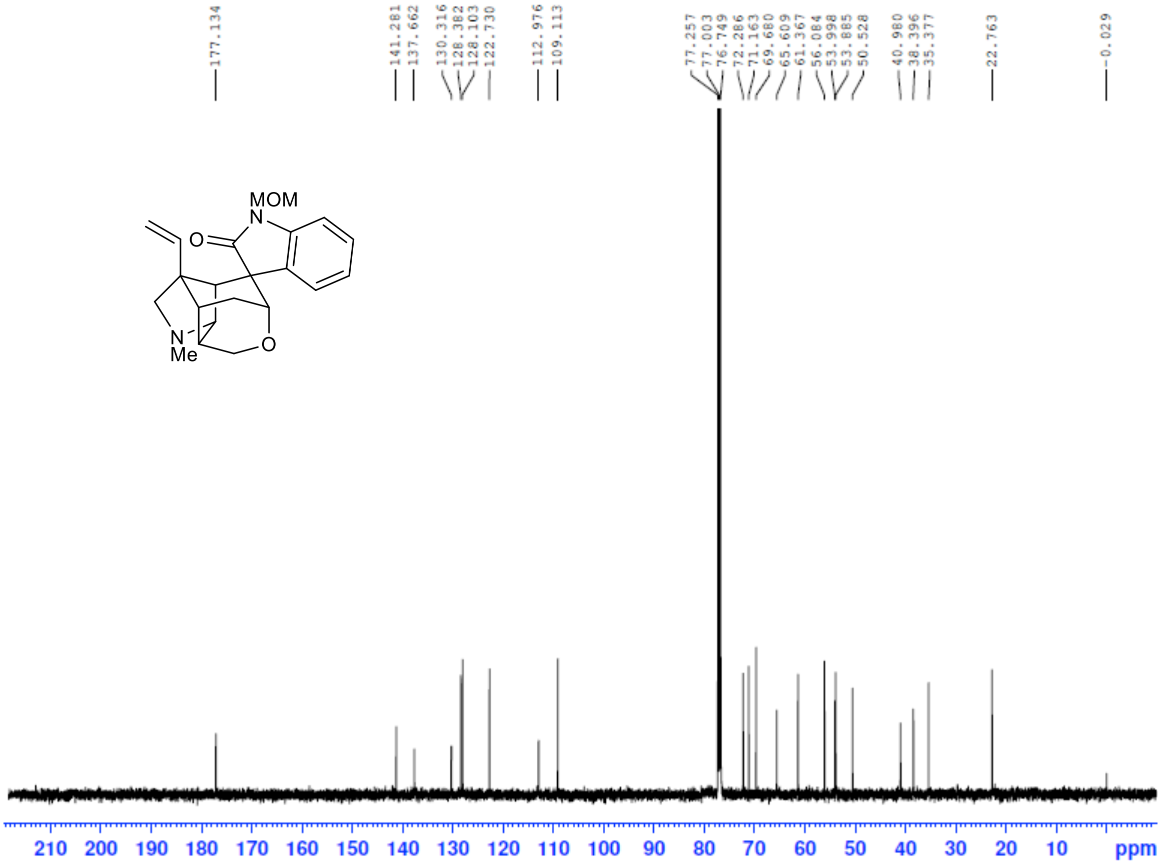

Supplementary figure 13. ^{1}H and ^{13}C NMR spectra for substrate **13**

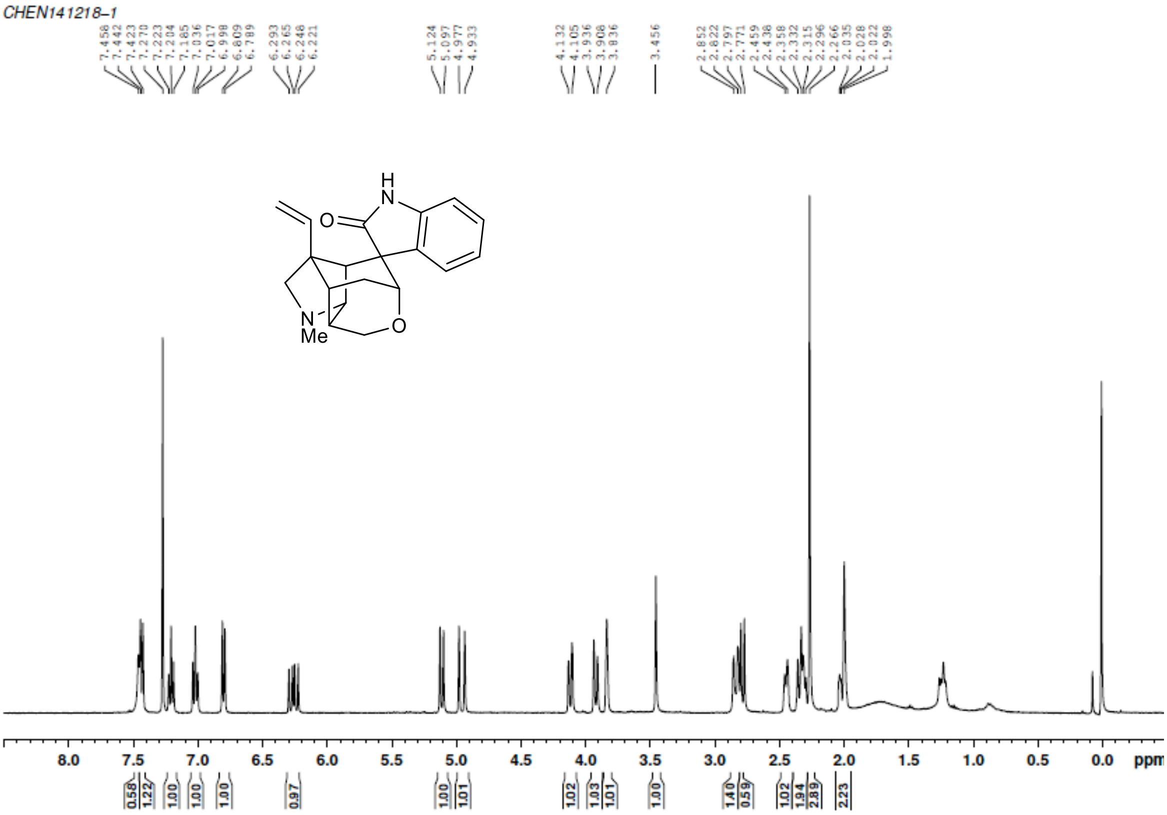

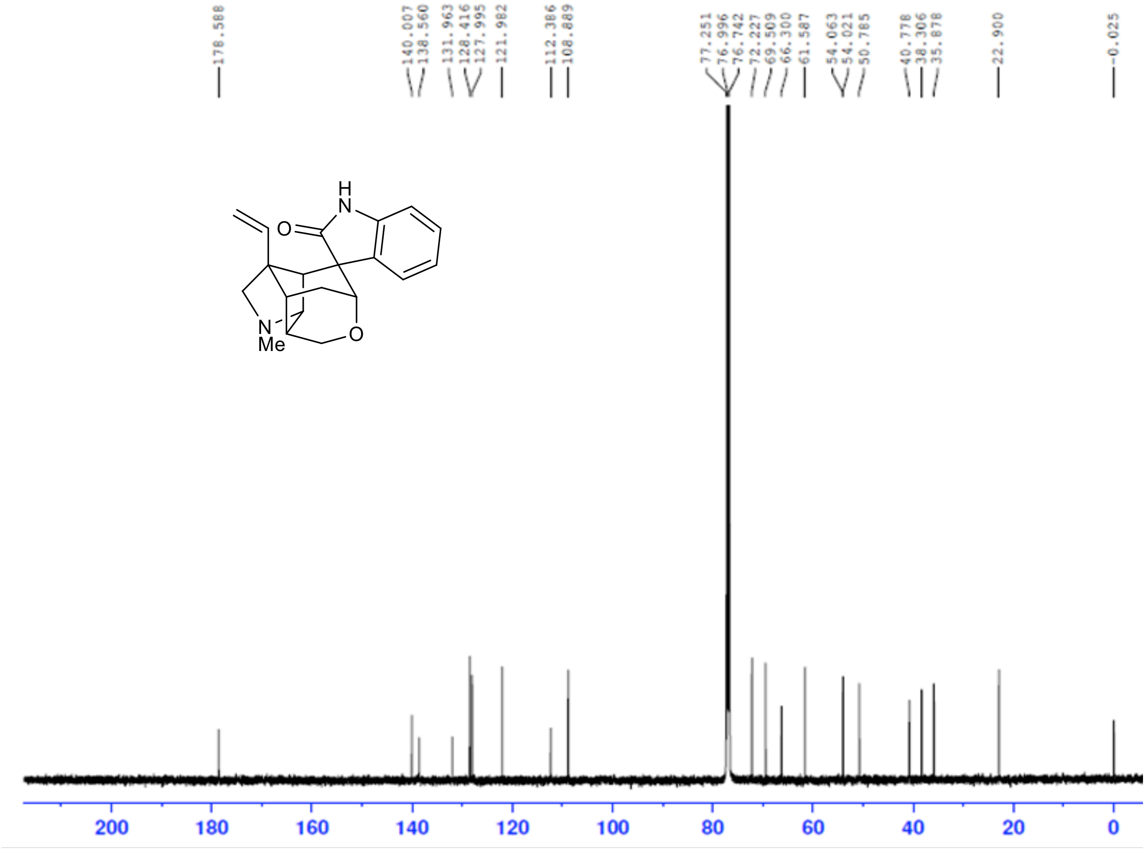

Supplementary figure 14. ^{1}H and ^{13}C NMR spectra for (+)-gelsemine

Nature (+)-gelsemine

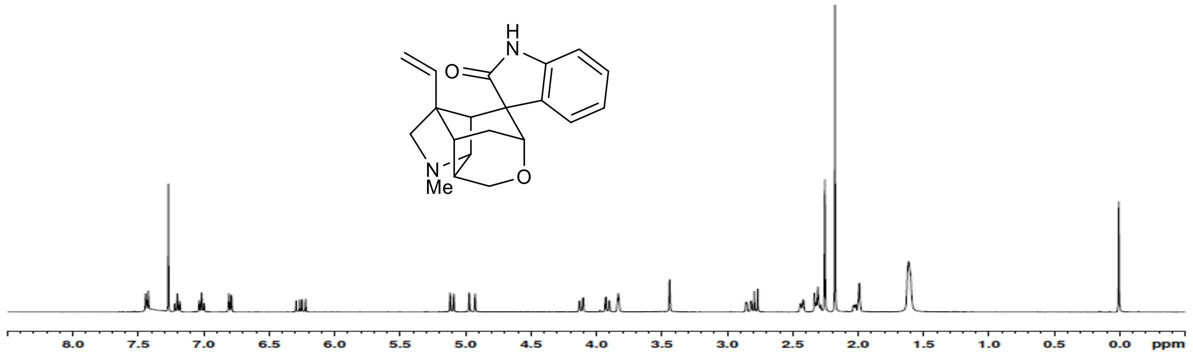

Our synthesis of (+)-gelsemine

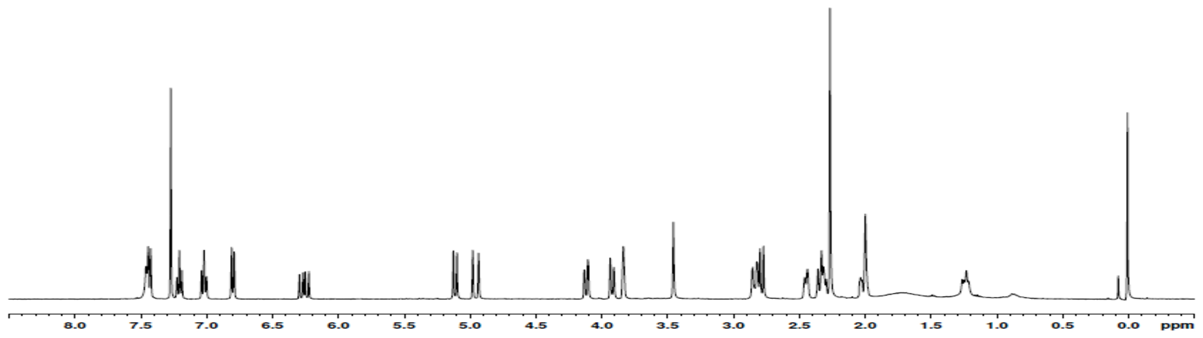

Supplementary figure 15. ^{1}H for nature (+)-gelsemine and our synthetic (+)-gelsemine

Empower software

进样综合报告 报告

用户名称： System　　项目名称： A2012_4_9

样品信息

样品名称:	chen-rac	采集者:	System
样品类型:	未知	采集时间:	2013-11-26 14:29:01
瓶号:	1	采集方法组:	zhaihbin
进样次数:	1	处理日期:	2013-11-26 15:11:58
进样体积:	5.00 ul	处理方法:	chen rac
运行时间:	60.0 Minutes	通道名称:	WIn Ch1
样品组名称:		处理通道注释:	PDA 214.0 纳米

自动标尺色谱图

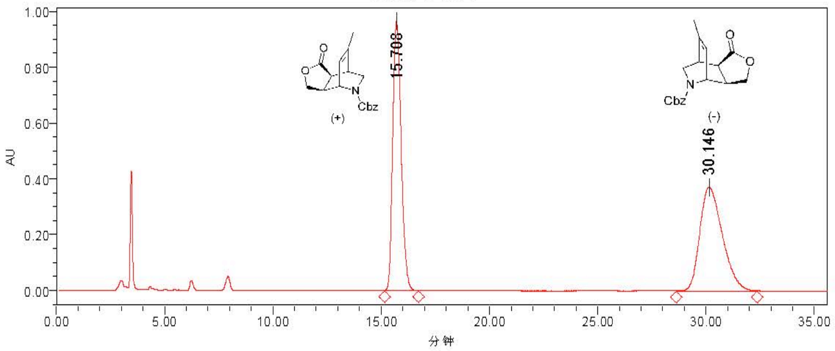

SampleName chen-rac; Vial 1; Injection 1; Channel W2996 ; Date Acquired 2013-11-26 14:29:01

处理通道: **PDA 214.0** 纳米

	处理通道	保留时间 (分钟)	面积	% 面积	峰高
1	PDA 214.0 纳米	15.708	26214129	49.06	968634
2	PDA 214.0 纳米	30.146	27223909	50.94	373424

Empower software

进样综合报告 报告

用户名称： System　　项目名称： A2014

样品信息

样品名称:	chen140103-1.2g	采集者:	System
样品类型:	未知	采集时间:	2014-1-3 12:25:43
瓶号:	1	采集方法组:	zhaihbin2014
进样次数:	3	处理日期:	2014-1-3 13:24:47
进样体积:	5.00 ul	处理方法:	chen140103 12g
运行时间:	50.0 Minutes	通道名称:	WIn Ch2
样品组名称:		处理通道注释:	PDA 210.4 纳米

自动标尺色谱图

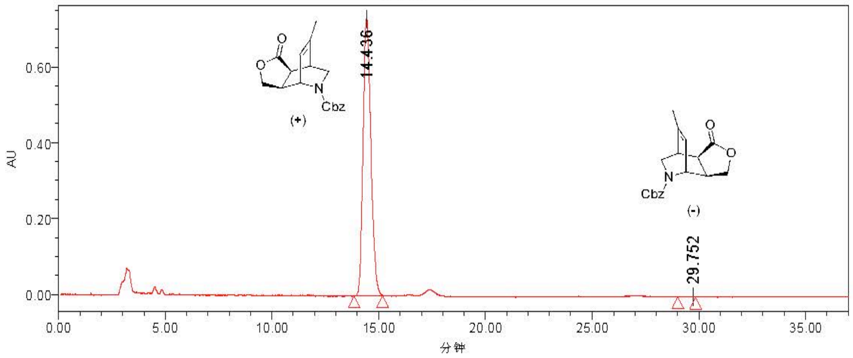

SampleName chen 140103-1.2g; Vial 1; Injection 3; Channel W2996 ; Date Acquired 2014-1-3 12:25:43

处理通道: **PDA 210.4** 纳米

	处理通道	保留时间 (分钟)	面积	% 面积	峰高
1	PDA 210.4 纳米	14.436	18648901	99.87	731555
2	PDA 210.4 纳米	29.752	24484	0.13	569

Supplementary figure 16. HPLC for compound **3:**

Supplementary Methods

General Information

All reagents were reagent grade and used without purification unless otherwise noted. All reactions involving air or moisture sensitive reagents or intermediates were performed under an inert atmosphere of argon in glassware that was oven dried. Reaction temperatures referred to the temperature of the cooling/heating bath. Chromatography was performed using forced flow (flash chromatography) of the indicated solvent system on 230-400 mesh silica gel (Silicycle flash F60) unless otherwise noted. ^{1}H NMR and ^{13}C NMR spectra were recorded on a Bruker AV-400 or 500 MHz spectrometer. Chemical shifts were referenced to the deuterated solvent (e.g., for $CDCl_3$, δ = 7.27 ppm and 77.0 ppm for ^{1}H and ^{13}C NMR, respectively) and reported in parts per million (ppm, δ) relative to tetramethylsilane (TMS, δ = 0.00 ppm). Coupling constants (*J*) were reported in Hz and the splitting abbreviations used were: s, singlet; d, doublet; t, triplet; q, quartet; m, multiplet; comp, overlapping multiplets of magnetically non-equivalent protons; br, broad; app, apparent. Reactions were monitored using thin-layer chromatography (TLC) carried out on 0.25 mm E. Merck silica gel plates (60F-254) using UV light as visualizing agent or an ethanolic solution of phosphomolybdic acid, cerium sulfate and heat as developing agents. Optical rotations were measured on a Perkin Elmer 341 polarimeter. Enantiomeric ratios were determined using chiral HPLC using a chiralpak AD-H (Amylose tris (3,5-dimethylphenylcarbamate)coated on 5μm silica-gel) with hexane and i-PrOH as eluents. Tetrahydrofuran, benzene, toluene and diethyl ether were distilled from Na and diphenylketone. Methylene chloride (DCM), N,N-diisopropylethylamine(DIPEA), and triethylamine were distilled from calcium hydride while methanol was distilled from dry magnesium turnings immediately before use.

General procedure for preparation of substrates

Procedure for synthesis of (+) - 3a

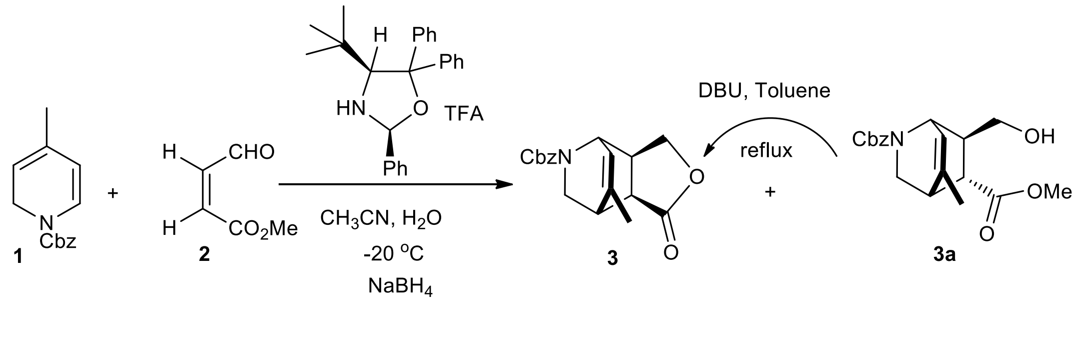

Catalyst salt[1,2] (10.2 mg, 0.02 mmol) was added to a solution of **2**[3,4] (50 mg, 0.42 mmol) in 1 mL CH_3CN and 52 μL H_2O at -20 °C. After the resulting mixture was stirred for 20 min, **1**[5,6] (50 mg, 0.21 mmol) was added dropwise to the reaction mixture during 20 min. The reaction mixture was stirred at -20 °C until no starting material remained as monitored by using TLC analysis (about 36 h) before $NaBH_4$ (17 mg, 0.42 mmol) was added to the reaction mixture at -20 °C. After the reaction was completed, the solvent was removed *in vacuo* and the residue

was diluted with EtOAc (10 mL) and H_2O (5 mL). The layers were separated and the aqueous phase was extracted with EtOAc (3 × 5 mL). The combine organic phases were washed with brine (2 × 5 mL), dried over Na_2SO_4, filtered and concentrated. The residue was purified by using silica gel column chromatography (PE/Acetone 10:1-4:1) to afford **3** (32 mg, 47%, >99% ee;); HPLC (Chiralcel® AD-H column; 85:15 Hexane/Isopropanol; flow rate = 1 mL/min; t_1 = 15.71 min (major), t_2 = 30.15 min (minor)) as a white solid and **3a** (22 mg, 30%) as a pale yellow oil.

To a solution **3a** (32 mg, 0.09 mmol) in anhydrous toluene (1 mL) was added DBU (0.2 mL). The reaction mixture was refluxed for 24 h before it cooled to room temperature. The reaction mixture was diluted with EtOAc (10 mL) and aqueous NH_4Cl (5 mL). The layers were separated and the aqueous phase was extracted with AcOEt (3 × 5 mL). The combined organic phases were washed with brine, dried over Na_2SO_4, filtered and then concentrated. The residue was purified by using silica gel column chromatography (PE/Acetone 4:1) to afford **3** (28 mg, 97%) as a white solid.

3: mp 110.1 – 110.7 °C; $[\alpha]^{17}_D$= 101.0 ($CHCl_3$, c = 1); ^1H NMR (400 MHz, $CDCl_3$) δ 7.36 – 7.33 (m, 5H), 6.13 (d, *J* = 6.0 Hz, 0.6H), 6.08 (d, *J* = 5.6, 0.4H), 5.12 (d, *J* = 6.4, 2H), 4.81 (dd, *J* = 6.0, 3.6 Hz, 0.6H), 4.67 (dd, *J* = 6.0, 3.6 Hz, 0.4H), 4.35 – 4.27 (m, 1H), 4.79 (dd, *J* = 10, 4.4 Hz, 0.6H), 3.75 (dd, *J* = 9.6, 4.0 Hz, 0.4H), 3.34 (t, *J* = 8.8 Hz, 1H), 3.11 – 3.08 (m, 3H), 2.95 – 2.94 (m, 1H), 1.87 (s, 3H); ^{13}C NMR (100 MHz, $CDCl_3$) δ 177.2, 177.1, 154.9, 154.4, 144.9, 144.6, 136.5, 136.4, 128.5, 128.4, 128.2, 128.1, 128.0, 127.8, 123.6, 123.1, 69.4, 69.3, 67.2, 67.0, 48.3, 47.9, 45.0, 44.6, 41.4, 39.3, 39.1, 38.3, 38.1, 20.6; HRMS (ESI) exact mass calculated for $[M+Na]^+$ ($C_{18}H_{19}NO_4Na^+$) required *m*/*z* 336.1212, found *m*/*z* 336.1206.

3a: $[\alpha]^{17}_D$= 74.0 ($CHCl_3$, c = 1); ^1H NMR (400 MHz, $CDCl_3$) δ 7.35 – 7. 27 (m, 5H), 6.03 (d, *J* = 1.6 Hz, 0.6H), 5.98 (d, *J* = 5.6 Hz, 0.4H), 5.17 – 5. 05 (m, 2H), 4.78 (dd, *J* = 6.0, 2.4 Hz, 0.6H), 4.70 (dd, *J* = 6.4, 2.8 Hz, 0.4 H), 3.71 (d, *J* = 4.0 Hz, 3H), 3.47 – 3.30 (m, 3H), 3. 02 – 2.96 (m, 1H), 2.87 (s, 1H), 2.72 – 2.70 (m, 1H), 2.40 (brsm, 0.6H), 2.22 (brsm, 0.4H), 2. 07 – 2.04 (m, 1H), 1.88 (s, 3H); ^{13}C NMR (100 MHz, $CDCl_3$) δ 173.9, 173.7, 154.9, 154.5, 143.6, 143.4, 143.3, 136.8, 136.7, 128.4, 128.3, 127.8, 127.7, 127.6, 124.3, 124.0, 66.8, 65.2, 65.1, 52.2, 52.1, 48.0, 47.7, 45.5, 42.8, 42.7, 42.6, 42.4, 38.9, 38.6, 19.3; HRMS (ESI) exact mass calculated for $[M+Na]^+$ ($C_{19}H_{23}NO_5Na^+$) required *m*/*z* 368.1474, found *m*/*z* 368.1468.

Procedure for synthesis of 4

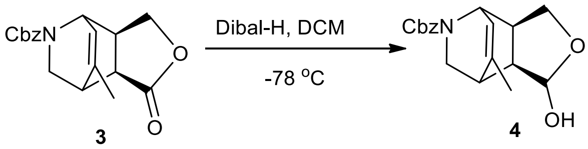

3 4

Dibal-H (1.0 mL, 1.5M, 1.45 mmol) was added dropwise to a solution of **3** (380 mg, 1.2 mmol) in anhydrous DCM (6.0 mL) at -78 °C under Ar. The reaction mixture was stirred for 3 h at -78 °C, at which time it was diluted with DCM (10 mL) and slowly quenched with a saturated aqueous solution of Rochelle's salt. The layers were separated and the aqueous phase was extracted with DCM (3 × 5 mL). The combined organic phases were washed with 1 M HCl, H_2O and brine, dried (Na_2SO_4), filtered and concentrated. The residue was purified

by using silica gel chromatography (PE/Acetone 2:1) to provide hemiacetal **4** (355 mg, 93%) as a white solid; mp 135.8 – 136.0 °C; $[\alpha]^{20}_{D}$ = 97.0 ($CHCl_3$, c = 1); ^{1}H NMR (400 MHz, $CDCl_3$) δ 7.36 – 7.33 (m, 5H), 6.04 (dd, *J* = 4.4, 1.6 Hz, 0.6H), 6.02 (dd, *J* = 7.2, 5.6 Hz, 0.4H), 5.08 (t, *J* = 10.4 Hz, 3H), 4.61 - 4.59 (m, 1H), 3.94 (q, *J* = 17.6, 9.2 Hz, 1H), 4.47 (ddd, *J* = 13.6, 7.2, 4.4 Hz, 1 H), 3.32 (dd, *J* = 7.2, 5.6 Hz, 0.6H), 3.23 (dd, *J* = 10.4, 2.0 Hz, 0.4H), 2.92 (ddd, *J* = 13.2, 10.4, 2.8 Hz, 1H), 2.82 – 2.79 (m, 2H), 2.44 (dd, *J* = 2.4, 1.2 Hz, 1H), 1.85 (s, 0.6H), 1.84 (s, 0.4); ^{13}C NMR (100 MHz, $CDCl_3$) δ 155.1, 154.7, 142.6, 142.2, 136.8, 136.7, 128.5, 128.4, 128.0, 127.9, 127.8, 127.7, 123.6, 123.1, 102.2, 69.6, 69.5, 66.9, 66.8, 49.5, 49.4, 48.6, 48.2, 46.1, 45.8, 43.3, 43.1, 38.8, 38.6, 21.3, 21.2; HRMS (ESI) exact mass calculated for $[M+Na]^+$ ($C_{18}H_{21}NO_4Na^+$) required *m/z* 338.1368, found *m/z* 338.1363.

Procedure for synthesis of acetal 5a and 5b

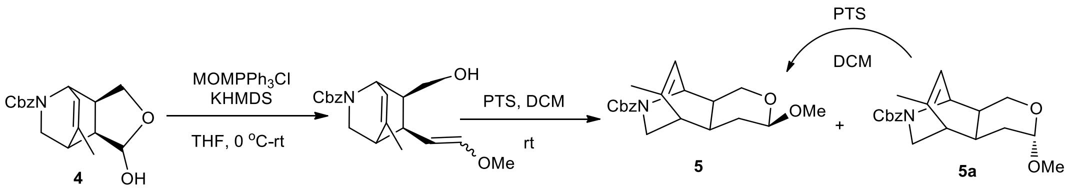

KHMDS (5.0 mL, 1 M, 5.0 mmol) was slowly added to a solution of $MOMPPh_3Cl$ (1.5 g, 4.4 mmol) in THF (10 mL) at 0 °C under Ar. The reaction mixture was allowed to warm to room temperature and stirred for 2.5 h before cooled to 0 °C, **4** (337 mg, 1.07 mmol) in 10 mL THF was slowly added to the reaction mixture, The reaction was aged for a period of 3 h and then diluted with EtOAc (10 mL) and quenched with saturated aqueous NH_4Cl. The layers were separated and aqueous phase was extracted with EtOAc (3 × 5 mL), the combined organic layers were washed with saturated NH_4Cl and brine, then dried (Na_2SO_4), filtered and concentrated to furnish a residue which was dissolved in 15 mL anhydrous DCM and trimethyl orthoformate (234 μL, 10.7 mmol) and PTS (20 mg, 0.01 mmol) was added at rt. The reaction was aged for a period of 24 h , and was then diluted with DCM (20 mL) and quenched with saturated aqueous $NaHCO_3$. The layers were separated and aqueous phase was extracted with DCM (3 × 5 mL). The combined organic layers were washed with brine, dried (Na_2SO_4), filtered and concentrated. The residue was purified by using silica gel chromatography (PE/Acetone 20:1- 8:1) to afford two isomers **5** and **5a** (330 mg, 90%) as pale yellow oil in a 13:1 ratio.

5: $[\alpha]^{17}_{D}$ = 6.0 ($CHCl_3$, c = 1); ^{1}H NMR (400 MHz, $CDCl_3$) δ 7.35 – 7.27 (m, 5H), 6.00 (d, *J* = 6.0 Hz, 0.6H), 5.94 (d, *J* = 6.0 Hz, 0.4H), 5.12 – 5.10 (m, 2H), 4.69 (dd, *J* = 9.2, 5.2 Hz, 1H), 4.46 (dd, *J* = 6.0, 2.4 Hz, 0.6H), 4.34 (dd, *J* = 6.0, 2.4Hz, 0.4H), 3.56 (dd, *J* = 11.2, 6.0 Hz, 0.6H), 3.53 (dd, *J* = 9.6, 6.0 Hz, 0.4H), 3.35 (s, 3 H), 3.30 (td, *J* = 7.2, 2.0 Hz, 1H), 3.21 (t, *J* = 12 Hz, 1 H), 3.13 – 3.09 (m, 1H), 2.45 – 2.41 (m, 2H), 2.08 -1.99 (m, 1H), 1.89 – 1.80 (m, 4H), ^{13}C NMR (100 MHz, $CDCl_3$) δ 154.7, 154.4, 143.2, 142.8, 136.9, 136.8, 128.4, 128.3, 127.9, 127.8, 127.6, 123.5, 123.0, 99.1, 99.0, 66.7, 66.6, 60.0, 54.7, 54.6, 47.0, 46.6, 46.4, 46.1, 41.2, 40.9, 40.3, 40.2, 30.7, 30.6, 30.3, 21.6, 21.5; HRMS (ESI) exact mass calculated for $[M+Na]^+$ ($C_{20}H_{25}NO_4Na^+$) required *m/z* 366.1681, found *m/z* 366.1676.

5a: $[\alpha]^{18}_{D}$ = 78.0 ($CHCl_3$, c = 1); ^{1}H NMR (400 MHz, $CDCl_3$) δ 7.35 – 7.33 (m, 5H), 5.99 (dd,

J = 4.4, 1.6 Hz, 0.6H), 5.93 (d, J = 6.4 Hz, 0.4H), 5.12 – 5. 09 (m, 2H), 4.75 (s, 1H), 4.46 (dd, J = 6.0, 2.8 Hz, 0.6H), 4.34 (dd, J = 6.0, 2.8 Hz, 0.4H), 3.68 (dd, J = 11.2, 6.0 Hz, 0.6H), 3.65 (dd, J = 6.8, 1.6 Hz, 0.4H), 3.36 (s, 3H), 3.36 – 3.33 (m, 1H), 3.22 – 3.14 (m, 1H), 3.07 – 3.03 (m, 1H), 2.75 – 2.62 (m, 1H), 2.39 (t, J = 2.04 Hz, 0.4H), 2.34 (t, J = 2.04 Hz, 0.6H), 2.32 – 2.24 (m, 1H), ^{13}C NMR (100 MHz, $CDCl_3$) δ 155.0, 154.6, 142.6, 142.2, 137.0, 136.9, 128.4, 128.3, 127.9, 127.84, 127.81, 127.7, 124.2, 123.8, 98.5,98.4, 66.8, 66.7, 62.3, 62.2, 54.9, 54.8, 48.0, 47.6, 47.5, 47.2, 41.0, 40.8, 39.9, 39.7, 30.1, 27.8, 27.7, 21.43, 21.41; HRMS (ESI) exact mass calculated for $[M+Na]^+$ ($C_{20}H_{25}NO_4Na^+$) required m/z 366.1681, found m/z 366.1676.

Procedure for synthesis of aldehyde 6 and hydroxyl-ketone 6

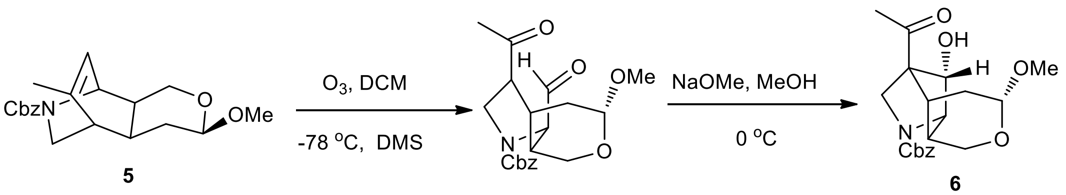

To a solution of **5** (150 mg, 0.44 mmol) in DCM (5 mL) was cooled to -78 °C and a stream of ozone was bubbled through the solution until a reaction mixture was turned into a blue. Argon was bubbled through the reaction mixture for 10 min. The mixture was treated with Me_2S (0.1 mL), warmed to room temperature, and concentrated *in vacuo* to give aldehyde as a residue which was diluted with anhydrous MeOH (44 mL) and cooled to 0 °C. It was then treated with NaOMe (7.1 mg. 0.13 mmol). The reaction mixture was stirred at 0 °C for 48 h and quenched by addition of NH_4Cl (6 mg, 0.17 mmol), warmed to room temperature and the solvent was removed *in vacuo* , the residue was diluted with EtOAc (20 mL) and H_2O, the layers were separated and aqueous layer was extracted with EtOAc (3 × 5 mL), the combined organic layers were washed with brine, dried over Na_2SO_4, filtered and concentrated, the residue was purified by using flash chromatography (PE/Acetone 8:1-4:1) afforded hydoxy-ketone **6** (99 mg, 60%) as a white solid; mp 48.5 – 49.5 °C; $[\alpha]^{19}_D$ = -112.2 ($CHCl_3$, c = 1); ^1H NMR (400 MHz, CD_3OD) δ 7.39 – 7.32 (m, 5H), 5.15 (s, 2H), 4.64 (dd, J = 8.8, 5.2 Hz, 1H), 4.53 (d, J = 5.6 Hz, 1H), 3.84 (d, J = 13.6 Hz, 1H), 3.77 (t, J = 9.2 Hz, 1H), 3.68 (dd, J = 11.2, 6.0 Hz, 0.6H), 3.64 (dd, J = 12.0, 6.4 Hz, 0.4H), 3.53 – 3.42 (m, 2H), 3.33 (s, 3H), 2.32 – 2.24 (m, 2H), 2.20 (d, J = 8.0 Hz, 3H), 1.69 – 1.65 (m, 1H), 1.54 – 1.49 (m, 1H); ^{13}C NMR (100 MHz, CD_3OD) δ 208.3, 208.2, 156.9, 156.6, 138.1, 138.0, 129.5, 129.1, 128.9, 128.8, 99.6, 73.7, 73.3, 68.12, 68.10, 66.1, 65.6, 63.5, 63.2, 59.52, 59.49, 55.0, 52.1, 52.0, 42.4, 42.1, 38.8, 29.3, 29.28, 29.27, 28.34, 28.32; HRMS (ESI) exact mass calculated for $[M+Na]^+$ ($C_{20}H_{25}NO_6Na^+$) required m/z 398.1580, found m/z 398.1583.

Procedure for synthesis of dihydroxy 7

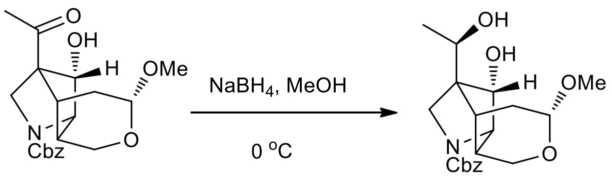

6 → 7

$NaBH_4$ (12 mg, 0.32 mmol) was slowly added into a solution of **6** (99 mg, 0.26 mmol) in dry MeOH (3 mL) at 0 °C, after being stirred for 30 min, the reaction was quenched by addition of H_2O, and the solvent was removed under vacuum. The residue was diluted with EtOAc (10 mL) and H_2O, the layers were separated and the aqueous was extracted with EtOAc (3 × 5 mL), and combined organic layers were washed with brine, dried over Na_2SO_4, filtered and concentrated. The residue was purified by using silica gel chromatography (PE/Acetone 4:1) to afford **7** (92 mg, 93%) as a white solid. mp 59.5 – 60.4 °C; $[\alpha]^{17}_D$ = -196.7 ($CHCl_3$, c = 0.3); ^{1}H NMR (400 MHz, CD_3OD) δ 7.38 – 7.31 (m, 5H), 5.17 – 5.14 (m, 2H), 6.64 (td, *J* = 4.8, 1.6 Hz, 1H), 4.22 (s, 1H), 4.03 (dt, *J* = 12.4, 6.0 Hz, 1H), 3.79 (d, *J* = 16.8 Hz, 1H), 3.69 (dd, *J* = 11.2, 6.0 Hz, 0.6H), 3.65 (dd, *J* = 11.2, 6.4 Hz, 0.4H), 3.50 (dt, *J* = 16.4, 9.6 Hz, 2H), 3.36 (s, 1H), 3.03 (dd, *J* = 20.4, 10.0 Hz, 1H), 2.20 – 2.02 (m, 3H), 1.21 (qd, *J* = 13.6, 9.6 Hz, 1H), 1.26 (dd, *J* = 8.8, 6.4 Hz, 3H); ^{13}C NMR (100 MHz, CD_3OD) δ 157.9, 157.7, 139.1, 139.0, 130.4, 130.0, 129.68, 129.66, 101.4, 74.6, 74.1, 68.82, 68.81, 67.7, 67.6, 64.8, 64.5, 60.9, 60.8, 58.7, 58.2, 55.9, 53.5, 53.3, 42.6, 42.3, 37.6, 29.2, 20.4; HRMS (ESI) exact mass calculated for $[M+Na]^+$ ($C_{20}H_{27}NO_6Na^+$) required *m*/*z* 400.1736, found *m*/*z* 400.1734.

Procedure for synthesis compound 8

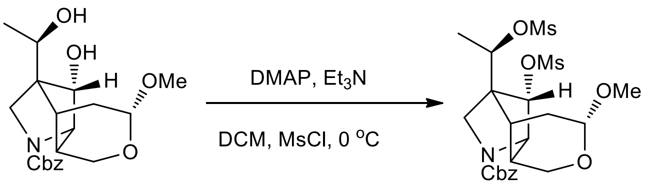

7 → 8

To a solution of **7** (93 mg, 0.25 mmol) in DCM (3 mL) was added DMAP (90 mg, 0.75 mmol). Et_3N (180 μL, 1.25 mmol), and stirred for 10 min at 0 °C, then MsCl (50 μL, 0.63 mmol) was slowly added to the reaction mixture at 0 °C, The reaction was aged for a period of 30 min and then diluted with DCM (10 mL) before quenched with saturated aqueous NH_4Cl, the layers were separated and the aqueous layer was extracted with DCM (10 mL), the combined organic layers were washed with brine, dried over Na_2SO_4, filtered and concentrated. The residue was purified by using silica gel chromatography (PE/Acetone 4:1) to afford **8** (131 mg, 100%) as a white solid. mp 81.5 – 82.3 °C; $[\alpha]^{17}_D$ = -40.0 ($CHCl_3$, c = 0.9); ^{1}H NMR (400 MHz, $CDCl_3$) δ 7.36 -7.31 (m, 5H), 5.18 – 5.09 (m, 2H), 5.01 (dd, *J* = 14.0 , 6.8 Hz, 1H), 4.94 (d, *J* = 13.2 Hz, 1H), 4.68 – 4.61 (m, 1H), 4.31 (d, *J* = 13.6 Hz, 1H), 3.75 (dd, *J* = 11.6, 6.8 Hz, 0.6H), 3.71 (dd, *J* = 11.6, 6.8 Hz, 0.4H), 3.55 – 3.46 (m, 2H), 3.37 (s, 3H), 3.12 (d, *J* = 12.6 Hz, 1H), 3.08 (s, 3H), 2.97 (d, *J* = 10.4 Hz, 3H), 2.47 – 2.32 (m, 1H),

2.17 - 2.14 (m, 2H), 1.72 – 1.59 (m, 1H), 1.49 (d, J = 6.4 Hz, 3H); ^{13}C NMR (100 MHz, $CDCl_3$) δ 154.5, 154.1, 136.2, 136.1, 128.50, 128.46, 128.2, 128.10, 128.03, 127.99, 98.02, 98.0, 78.2, 77.85, 75.1, 74.8, 67.3, 67.2, 60.0, 59.5, 57.8, 57.7, 56.3, 55.6, 54.8, 52.8, 52.5, 40.7, 40.3, 39.02, 39.01, 38.8, 38.5, 34.1, 34.0, 27.23, 27.17, 17.7, 17.6; HRMS (ESI) exact mass calculated for $[M+Na]^+$ ($C_{22}H_{31}NO_{10}S_2Na^+$) required m/z 556.1287, found m/z 556.1282.

Procedure for synthesis 9

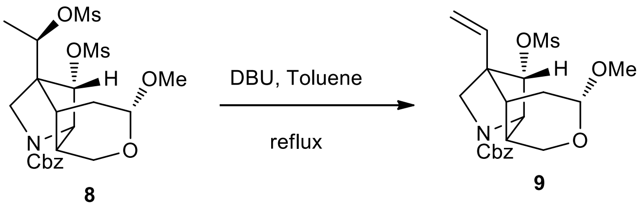

To a solution of **8** (131 mg, 0.24 mmol) in anhydrous toluene (5 mL) was added DBU (1 mL) at room temperature. The reaction mixture was warmed to reflux for 36 h and then allowed to cooled to rt. The reaction mixture was diluted with EtOAc (15 mL) and quenched with saturated aqueous NH_4Cl, the layers were separated and aqueous phase was extracted with EtOAc (3 × 5 mL), the combined organic phases were dried (Na_2SO_4), filtered and concentrated. The residue was purified by using silica gel chromatography (PE/Acetone 8:1) to afford **9** (91 mg, 85%) as a white solid. mp 46.6 – 47.3; $[\alpha]^{20}_D$= -90.0 ($CHCl_3$, c = 1); ^{1}H NMR (400 MHz, $CDCl_3$) δ 7.37 – 7.35 (m, 5H), 5.86 (dq, J = 17.2, 11.2, 5.6 Hz, 1H), 5.34 (dd, J = 11.2, 2.4 Hz, 1H), 5.22 – 5.12 (m, 3H), 4.98 (d, J = 8.4 Hz, 1H), 4.66 (dd, J = 8.8, 5.2 Hz, 1H), 4.25 (d, J = 17.6 Hz, 1H), 3.78 – 3.66 (m, 2H), 3.37 (s, 3H), 3.28 (dd, J = 9.6, 6.8 Hz, 1H), 2.90 (d, J = 10.8 Hz, 3H), 2.49 – 2.33 (m, 1H), 2.18 – 1.95 (m, 2H), 1.51 – 1.40 (m, 1H); ^{13}C NMR (100 MHz, $CDCl_3$) δ 154.5, 154.4, 136.5, 136.4, 130.98, 130.93, 128.53, 128.51, 128.2, 128.04, 127.95, 119.3, 119.2, 98.2, 98.1, 80.4, 80.1, 67.12, 67.07, 60.6, 60.1, 58.03, 57.98, 55.2, 54.9, 54.8, 54.5, 53.0, 52.8, 40.6, 40.2, 38.8, 38.6, 37.5, 27.79, 27.75; HRMS (ESI) exact mass calculated for $[M+Na]^+$ ($C_{21}H_{27}NO_7SNa^+$) required m/z 460.1406, found m/z 460.1402.

Procedure for synthesis of 10

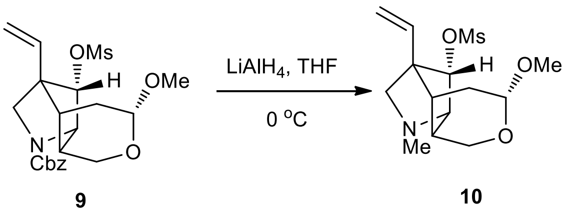

To a solution of **9** (37 mg, 0.08 mmol) in anhydrous THF (1.0 mL) was added $LiAlH_4$ (3.6 mg, 0.1 mmol) at 0 °C under Ar. The reaction mixture was stirred at 0 °C until the start material was disappeared. Added 10% NaOH quenched the reaction and fileted by using celite and washed with MeOH. The combined organic phases were concentrated under vacuum and the residue was purified by using silica gel chromatography (DCM/MeOH 50:1) to afford **10** (23 mg, 83%) as a whiteness oil. $[\alpha]^{20}_D$ = -79.0 ($CHCl_3$, c = 1); ^{1}H NMR (400 MHz, $CDCl_3$) δ 5.87 (dd, J = 17.6, 11.2 Hz, 1H), 5.27 (dd, J = 11.2, 1.2 Hz, 1 H), 5.13 (dd, J = 17.6, 1.2 Hz, 1 H), 4.95 (s, 1H), 4.66 (dd, J = 8.8, 5.2 Hz, 1H), 3.64 （dd, J = 11.6, 6.8 Hz,

1H），3.46 (t, J = 12.4 Hz, 1H), 3.37 (s, 3H), 3.15 (d, J = 9.2 Hz, 1H), 3.10 (s, 1H), 3.06 (s, 3H), 2.58 (d, J = 9.2 Hz, 1H), 2.52 (s, 3H), 2.51 – 2.45 (m, 1H), 1.97 – 1.83 (m, 2H), 1.46 – 1.37 (m, 1H); ^{13}C NMR (100 MHz, $CDCl_3$) δ 132.5, 118.2, 98.5, 82.9, 66.1, 61.5, 58.8, 55.7, 54.8, 40.8, 38.8, 37.8, 36.4, 27.9; HRMS (ESI) exact mass calculated for $[M+H]^+$ ($C_{14}H_{23}NO_5SH^+$) required *m/z* 318.1370, found *m/z* 318.1370.

Procedure for synthesis of 11

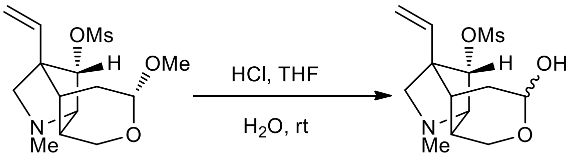

10 11

To a solution of **10** (25 mg, 0.08 mmol) in THF (1 mL) was added 2M HCl (1 mL). The reaction was aged for a period of 2 h and then diluted with $CHCl_3$ (2 mL) and quenched with saturated aqueous Na_2CO_3, the layers were separated and the aqueous was extracted with $CHCl_3$ (3 × 5 mL), the combined organic layers were washed with brine, dried over Na_2SO_4, filtered and concentrated. The residue was purified by using silica gel chromatography (DCM/CH_3OH 4:1) to afford **11** (23 mg, 97%) as white solid. ^{1}H NMR (400 MHz, $CDCl_3$) δ 5.92 – 5.83 (m, 1H), 5.33 – 5.29 (m, 1H), 5.18 – 5.12 (m, 1H), 5.04 (s, 1H), 4.50 (brsm, 1H), 3.80 (dd, J = 12.0, 5.6 Hz, 0.4H), 3.70 – 3.66 (m, 0.6H), 3.59 (t, J = 11.6 Hz, 0.6H), 3.47 (dd, J = 12.4, 7.6 Hz, 0.4H), 3.26 (s, 0.4H), 3.20 (s, 1H), 3.17 (s, 0.6H), 3.08 (s, 3H), 2.87 – 2.73 (m, 1H), 2.66 – 2.49 (m, 4H), 2.30 – 2.28 (m, 0.4H), 1.99 – 1.84 (m, 1H), 1.83 – 1.81 (m, 0.4H), 1.78 – 1.73 (m, 0.4H), 1.48 – 1.44 (m, 0.6H); ^{13}C NMR (100 MHz, $CDCl_3$) δ 132.1, 132.0, 118.7, 118.6, 91.8, 90.7, 83.0 ,82.6, 68.5, 66.4, 62.1, 61.3, 60.1, 59.0, 55.5, 54.6, 41.3, 40.9, 38.7, 37.9, 36.4, 35.8, 35.2, 28.9, 27.7; HRMS (ESI) exact mass calculated for $[M+H]^+$ ($C_{13}H_{21}NO_5SH^+$) required *m/z* 304.1213, found *m/z* 304.1217.

Procedure for synthesis of 12

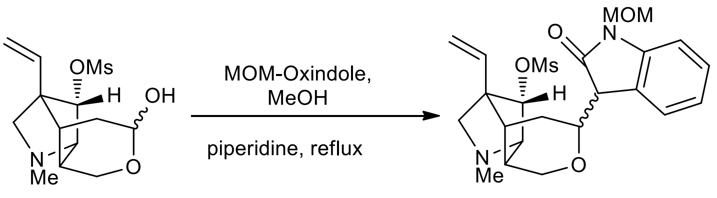

11 12

To a solution of **11** (23 mg, 0.08 mmol) in MeOH (1 mL) was added MOM-Oxindole (21 mg, 0.11 mmol), the reaction mixture was warmed to reflux for 8 h before cooled to room temperature. Then the reaction mixture was diluted with EtOAc (20 mL) and quenched with brine. The layers were separated and aqueous was extracted with EtOAc (3 × 5 mL), the combined organic layers were washed with brine, dried (Na_2SO_4), filtered and concentrated. The residue was purified by using silica gel chromatography (DCM/MeOH 100:1 - 20:1) to afford **12** (32 mg, 93%) as pale yellow solid. ^{1}H NMR (400 MHz, $CDCl_3$) δ 7.37 (d, J = 5.2

Hz, 0.2H), 7.35 – 7.27 (m, 1.8H), 7.14 – 7.09 (m, 1H), 7.03 (d, J = 8.0 Hz, 1H), 5.95 – 5.77 (m ,1H), 5.32 – 4.80 (m, 5H), 4.33 – 4.29 (m, 1H), 3.89 – 3.74 (m, 0.3H), 3.69 – 3.63 (m, 1.3H), 3.58 – 3.48 (m, 2H), 3.48 – 3.02 (m, 8H), 2.61 – 2.49 (m, 5H), 2.16 (s, 0.6H), 2.04 – 1.92 (m, 1H), 1.90 – 1.85 (m, 1H), 1.78 – 1.65 (m, 0.5H), 1.55 – 1.53 (m, 0.6H); ^{13}C NMR (100 MHz, $CDCl_3$) δ 176.1, 175.5, 143.1, 142.9, 142.8, 132.6, 132.5, 132.4, 128.5, 128.4, 128.3, 126.0, 126.0, 125.2, 125.0, 124.9, 124.2, 123.3, 123.0, 122.8, 118.3, 118.2, 109.5, 109.4, 109.3, 83.5, 83.4, 83.1, 82.9, 74.0, 73.5, 72.7, 71.1, 71.0, 67.6, 66.9, 66.5, 63.0, 62.5, 62.3, 62.0, 61.7, 61.6, 56.2, 56.1, 56.0, 55.7, 55.6, 55.0, 51.8, 51.4, 51.3, 50.5, 41.2, 40.8, 40.6, 40.5, 40.4, 38.8, 38.7, 38.2, 37.8, 36.2, 35.7, 35.4, 30.8, 29.6, 25.5, 23.8, 22.9; HRMS (ESI) exact mass calculated for $[M+H]^+$ ($C_{23}H_{30}N_2O_6SH^+$) required m/z 463.1897, found m/z 463.1901.

Procedure for synthesis of 13

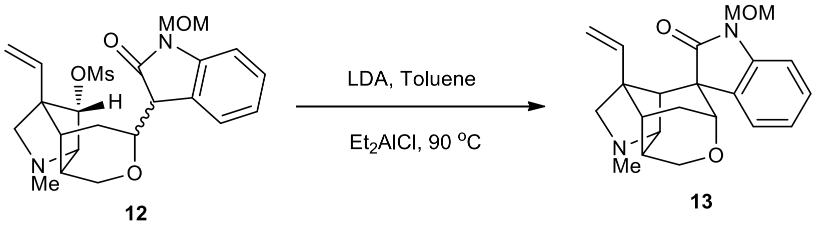

12 **13**

To a solution of **12** (15 mg, 0.03 mmol) in Toluene (1 mL) was added LDA (39 uL, 1 M, 0.04 mmol) at 0 °C, and stirred for 30 min at 0 °C, then Et_2AlCl (80 uL, 2 M, 0.15 momol) was added. The reaction mixture was warmed to 90 °C until the starting material was disappeared before it was cooled to room temperature, and diluted with EtOAc (15 mL), quenched with brine, the layers were separated and aqueous phase was extracted with EtOAc (3 × 5 mL), the combined organic phases were dried (Na_2SO_4), filtered and concentrated. The residue was purified by using silica gel chromatography (DCM/MeOH 100:1 – 50:1) to afford **13** (3.2 mg, 32%) as a amorphous solid. $[\alpha]^{20}_D$= 0.40 ($CHCl_3$, c = 1); ^{1}H NMR (500 MHz, $CDCl_3$) δ 7.44 (d, J = 7.7 Hz, 1H), 7.30 (t, J = 7.6 Hz, 1H), 7.10 (t, J = 7.6 Hz, 1H), 7.01 (d, J = 7.6 Hz, 1H), 6.21 (dd, J = 17.6, 10.8 Hz, 1H), 5.18 (d, J = 10.8 Hz, 1H), 5.16 (d, J = 10.8 Hz, 1H), 5.08 (d, J = 10.8 Hz, 1H), 4.99 (d, J = 17.6 Hz, 1H), 4.12 (dd, J = 11.2, 1.6 Hz, 1H), 3.97 (dd, J = 11.2, 1.6 Hz, 1H), 3.80 (s, 1H), 3.66 (s, 1H), 3.32 (s, 3H), 2.87 (dd, J = 14.4, 2.8 Hz, 1H), 2.77 (d, J = 10.8 Hz, 1H), 2.65 (d, J = 8.8 Hz, 2H), 2.42 (t, J = 7.2 Hz, 1H), 2.37 (s, 3H), 2.07 – 1.99 (m, 3H); ^{13}C NMR (125 MHz, $CDCl_3$) δ 177.13, 141.28, 137.66, 130.32, 128.38, 128.10, 122.73, 112.98, 109.11, 72.29, 71.16, 69.68, 65.61, 61.37, 56.08, 54.00, 53.89, 50.53, 40.98, 38.40, 35.38, 22.76; HRMS (ESI) exact mass calculated for $[M+H]^+$ ($C_{22}H_{26}N_2O_3H^+$) required m/z 367.2016, found m/z 367.2017.

Procedure for synthesis of (+)-gelsemine

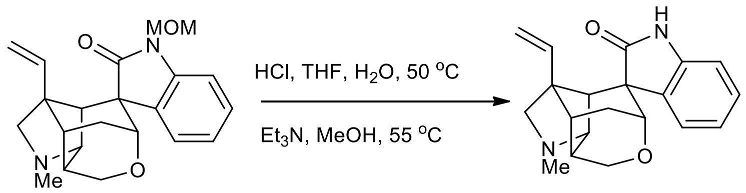

13 (+)-gelsemine

HCl (0.5 mL, 6 M) was added to a solution of **13** (3.2 mg, 8.8 μmol) in THF (0.5 mL). The resulting mixture was heated at 55 °C for 24 h and then cooled to rt, diluted with DCM (10 mL) and quenched with saturated aqueous $NaHCO_3$ (5 mL). The layers were separated and the aqueous layer was extracted with DCM (3 × 5 mL). The combined organic extracts were dried (Na_2SO_4), filtered, and concentrated. Methanol (1.5 mL) and Et_3N (14 μL, 88 μmol) were added to the residue and the resulting mixture was heated at 55 °C for 20 h. The reaction mixture was then allowed to cool to rt and concentrated. The residue was purified by using silica gel chromatography (MeOH/DCM 1:10) to afford 2.1 mg (70%) of gelsemine as a white solid. $[\alpha]^{20}_{D}$ = 10.0 ($CHCl_3$, c = 0.2); ^1H NMR (400 MHz, $CDCl_3$) δ 7.46 (brsm, 1H), 7.43 (d, J = 7.6 Hz, 1H), 7.20 (t, J = 7.6 Hz, 1H), 7.02 (t, J = 7.6 Hz, 1H), 6.89 (d, J = 7.6 Hz, 1H), 6.25 (dd, J = 18.0, 11.2 Hz, 1H), 5.11 (d, J = 10.8 Hz, 1H), 4.96 (d, J = 17.6 Hz, 1H), 4.12 (d, J = 10.8 Hz, 1H), 3.92 (d, J = 9.6 Hz, 1H), 3.84 (s, 1H), 3.46 (s, 1H), 2.85 – 2.77 (m, 2H), 2.78 (d, J = 8.0 Hz, 1H), 2.45 (d, J = 8.4 Hz, 1H), 2.36 – 2.29 (m, 2H), 2.27 (s, 3H), 2.04 – 2.00 (m, 2H); ^{13}C NMR (125 MHz, $CDCl_3$) δ 178.6, 140.0, 138.6, 131.96, 128.4, 127.99, 121.98, 112.4, 108.9, 72.2, 69.5, 66.3, 61.6, 54.06, 54.02, 50.8, 40.8, 38.3, 35.9, 22.9; HRMS (ESI) exact mass calculated for $[M+H]^+$ ($C_{20}H_{22}N_2O_2H^+$) required m/z 323.1754, found m/z 323.1747.

ORTEPS drawing of 8 from X-ray crystallographic analysis

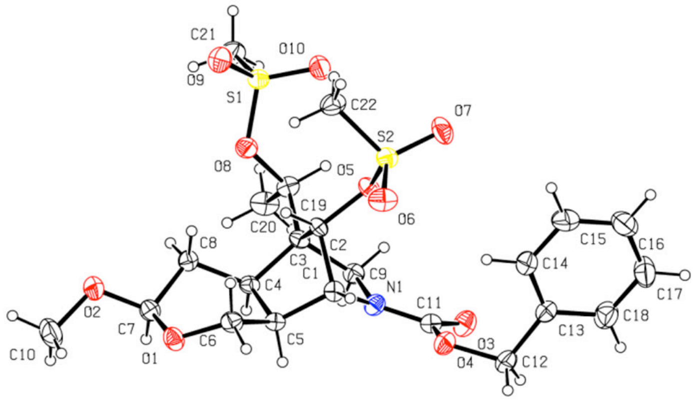

Supplementary Dataset 1

Compound **8** CCDC 1056043

Supplementary reference

[1] Nakano, H.; Osone, K.; Takeshita, M.; Kwon, E.; Seki, C.; Matsuyama, H.; Takano, N. & Kohan, Y. A novel chiral oxazolidine organocatalyst for the synthesis of an oseltamivir intermediate using a highly enantioselective Diels-Alder reaction of

1,2-dihydropyridine. *Chem. Commun*. **46**, 4827-4829 (2010).

[2] Kohari, Y.; Okuyama, Y.; Kwon, E.; Furuyama, T.; Kobayashi, N.; Otuki, T.; Kumagai, J.; Seki, C.; Uwai, K.; Dai, G.; Iwasa, T. & Nakano, H. Enantioselective Diels-Alder reaction of 1,2-dihydropyridines with aldehydes using β-amino alcohol organocatalyst. *J. Org. Chem.* **79**, 9500-9511 (2014)

[3] Iesce, M. R.; Cermola, F.; Guitto, A.; Giordano, F.; & Scarpati, R. Carbonyl Oxide Chemistry. 5. Nucleophilic Trapping Reaction with Ald - and Ketoximes. Synthesis of Hydroperoxy nitrones. *J. Org. Chem.* **61**, 8677-8680 (1996).

[4] Iesce, M. R.; Cermola, F.; Guitto, A.; Scarpati, R.; Graziano, M. L., Carbonyl Oxide Chemistry. 4. Novel Observations on the Behavior of 1-Methoxy-2, 3, 7-trioxabicyclo [2.2.1] hept-5-ene. *J. Org. Chem.* **60**, 5324-5327 (1995).

[5] Bayly, A. R.; White, A. J. P.; Spivey, A. C. Design and Synthesis of a Prototype Scaffold for Five-Residue α-Helix Mimetics. *Eur. J. Org. Chem*. **25**, 5566-5569 (2013).

[6] Li, H.; Cheng, B.; Boonnak, N.; padwa, A., An approach toward the alkaloid (±)-mersicarpine using a rhodium(II) carbenoid cyclization-cycloaddition cascade of an α- diazo dihydroindolinone. *Tetrahedron* **67**, 9829- 9836 (2011).
